# Trans-Saharan migratory patterns in *Vanessa cardui* and evidence for a southward leapfrog migration

**DOI:** 10.1016/j.isci.2024.111342

**Published:** 2024-11-08

**Authors:** Megan S. Reich, Sana Ghouri, Samantha Zabudsky, Lihai Hu, Mael Le Corre, Ivy Ng’iru, Dubi Benyamini, Daria Shipilina, Steve C. Collins, Dino J. Martins, Roger Vila, Gerard Talavera, Clément P. Bataille

**Affiliations:** 1 Department of Biology, University of Ottawa, Ottawa, ON, Canada; 2 Department of Earth and Environmental Sciences, University of Ottawa, Ottawa, ON, Canada; 3 Department of Archaeology, University of Aberdeen, Aberdeen, UK; 4 UMR 7209 - AASPE, Muséum national d'Histoire naturelle, Paris, France; 5 Mpala Research Centre, Nanyuki, Laikipia, Kenya; 6 School of Biosciences, Cardiff University, Cardiff, Wales, UK; 7 UK Centre for Ecology and Hydrology, Wallingford, Oxfordshire, UK; 8 The Israeli Lepidopterist Society, Beit Arye, Israel; 9 Department of Ecology and Genetics, Uppsala University, Uppsala, Sweden; 10 African Butterfly Research Institute, Nairobi, Kenya; 11 McGuire Center for Lepidoptera and Biodiversity, University of Florida, Gainesville, FL, USA; 12 Turkana Basin Institute, Stony Brook University, Stony Brook, NY, USA; 13 Institut de Biologia Evolutiva, CSIC - Universitat Pompeu Fabra, Barcelona, Catalonia, Spain; 14 Institut Botànic de Barcelona (IBB), CSIC - CMCNB, Barcelona, Catalonia, Spain

**Keywords:** Chemistry, Nature conservation, Ecology, Entomology

## Abstract

Some insects, such as the painted lady butterfly *Vanessa cardui*, exhibit complex annual migratory cycles spanning multiple generations. Traversing extensive seas or deserts is often a required segment of these migratory journeys. We develop a bioavailable strontium isoscape for Europe and Africa and then use isotope geolocation combining hydrogen and strontium isotopes to estimate the natal origins of painted ladies captured north and south of the Sahara during spring and autumn, respectively. Our findings reveal moderate migratory connectivity across the Sahara characterized by a broad-front, parallel migration. We also report evidence of a leapfrog migration, wherein early autumn migrants from higher latitudes cover greater distances southward than their late autumn counterparts. This work represents a major advancement in understanding insect migratory patterns and connectivity, particularly across extensive barriers, which is essential for understanding population dynamics and predicting the impacts of global change on insect-mediated ecosystem services.

## Highlights

• Hydrogen and strontium isotopes characterize *Vanessa cardui*’s trans-Saharan migrations
• Autumn *V. cardui* migrate in a broad-front, parallel pattern with moderate connectivity
• Isotope geolocation indicates a leapfrog migration pattern during the autumnal migration
• We demonstrate migratory linkages between the eastern Mediterranean and East Africa

## Graphical abstract

**Figure:**
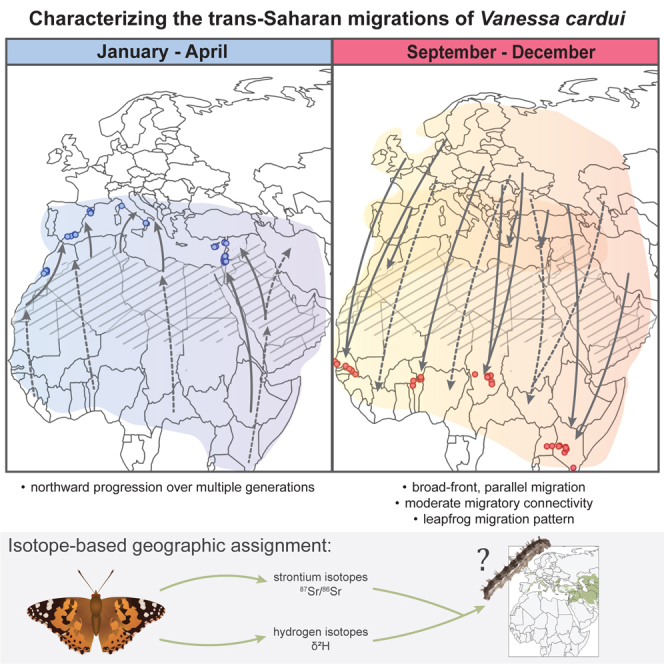

## Introduction

Multi-generational migrations equip insects with the capacity to respond swiftly to seasonal environmental changes, yet this very trait also renders them vulnerable to certain challenges. In contrast to migratory vertebrates, which typically complete round-trip migrations between overwintering and breeding grounds within a single generation, insects, with their shorter lifespans, require several generations to complete their annual migratory cycle, with each generation completing a single segment of the overall cycle.^1^ The spatial extent of suitable climatic and biotic conditions, largely determined by host plant availability, can change drastically throughout the year, prompting each generation to embark on migratory journeys in search of suitable habitats.^2^^,^^3^ However, many individual insects do not survive the journey, whereas others conclude their journeys in unsuitable habitats that cannot sustain the next generation, potentially leading to local- or regional-scale extirpation and a decline in population size. Fortunately, the range of suitable habitats for migratory insects tends to be broad, with reticular migratory patterns that provide redundancy and compensate for the impact of local bottlenecks.^4^ Although these reticular patterns are generally thought to enhance the resilience of populations to environmental perturbation,^5^^,^^6^ in the face of large-scale disturbances such as global climate change and habitat degradation, the population dynamics and migratory patterns of some species are nonetheless affected. For example, the monarch butterfly *Danaus plexippus* population in North America seems to be declining, with changing spring weather conditions playing an important role in the decline.^7^ Similarly, outbreaks of desert locust *Schistocerca gregaria* are influenced by the increasing frequency of extreme weather events,^8^ and changes to monsoon patterns are affecting the migration patterns of the brown planthopper *Nilaparvata lugens*, a devastating agricultural pest.^9^ Delineating predictable spatiotemporal linkages of insects across their migratory range is an imperative first step for predicting insect population dynamics and understanding how they will respond to global change.

Understanding migratory patterns and connectivity across natural barriers, such as seas and deserts, may be particularly important for insect population dynamics. Migratory journeys across barriers can be especially perilous, and large-scale losses can lead to population bottlenecks.^10^^,^^11^ The Sahara is the world’s third-largest desert and, for the migratory animals that traverse it, it constitutes at least a 1,000-kilometer journey through unsuitable habitat. The migratory connectivity of many bird species across the Sahara has been well explored (e.g.,^12^^,^^13^). These studies demonstrated that many of the bird species migrating across the Afro-Palearctic are facing population declines,^14^ illustrating how the understanding of migratory connectivity across biogeographic barriers and geopolitical borders is fundamental for proposing effective international conservation action (e.g.,^15^^,^^16^). Generally, insect migration remains understudied compared to research on migratory birds and mammals,^17^ and although there are growing indications that many insect species undertake trans-Saharan journeys, empirical evidence for crossings of the Sahara remains limited or ambiguous. As a result, the annual migrations of the painted lady butterfly *Vanessa cardui* stand out as the primary example of trans-Saharan insect migration and is an ideal model to study insect migration across natural barriers.

Over the past decade, regular seasonal migrations of the painted lady butterfly across the Sahara have been verified using a variety of techniques, including field observations, monitoring stations, isotope geolocation, ecological niche modeling, and pollen metabarcoding analyses.^3^^,^^18^^,^^19^^,^^20^^,^^21^^,^^22^^,^^23^ The Afro-Palearctic population of painted lady butterflies undergoes an annual migratory cycle of continuous movement and breeding spanning 8 to 10 overlapping generations.^23^ Painted ladies follow the oogenesis-flight syndrome, wherein there is an energetic trade-off between migration and reproduction.^24^^,^^25^^,^^26^ Thus, migration is expected to occur during the pre-reproductive phase. Painted lady adults live for about 3–6 weeks, mating multiple times during the reproductive phase and laying up to over 1,000 eggs that develop into adults over 23 to 45 days, depending on environmental conditions.^23^^,^^26^^,^^27^^,^^28^ Thus, the offspring of a single female can depart in several migratory waves, intermingling with older generations. Over multiple generations, painted ladies migrate as far north as Scandinavia during the summer, travel south to regions on both sides of the Sahara for the European winter (i.e., North Africa and sub-Saharan Africa), and then return to Europe in the spring.^19^^,^^21^^,^^22^ This recurring migration across the Sahara offers a unique opportunity to study insect migratory connectivity between well-differentiated faunistic regions and across a natural barrier.

There are multiple reasons why insect migration is understudied. For one, the multi-generational migratory cycle adds a layer of complexity to the study of insect migration, demanding investigation of each segment of the annual cycle. Moreover, understanding insect migratory patterns requires addressing both its spatial and temporal dimensions. Thus, large-scale collaborations are essential for investigating the international and intercontinental migrations of insects (e.g.,^19^^,^^29^). Insects are also difficult to track with techniques that are traditionally used to study vertebrates, like biologging technology (e.g., radiotelemetry, light loggers), because insects are small, abundant, and short-lived. Instead, intrinsic biomarkers, such as isotopes, have become quintessential tools for studying insect migration. The isotopic composition of the local environment is incorporated into developing tissues as the larval insect feeds.^30^^,^^31^ After metamorphosis, this local isotopic composition is largely preserved in the wings.^32^^,^^33^ Thus, the isotopic composition of a migrant butterfly’s wing can be measured and compared to a spatial model of isotopic variation to estimate the individual butterfly’s natal origin (i.e., isotope-based geographic assignment). Hydrogen isotope values (δ^2^H) have been used for insect geolocation for over 20 years^31^^,^^34^ and, due to global precipitation patterns, often act as a proxy for the latitude of origin.^35^ However, δ^2^H-based geographic assignment rarely provides adequate longitudinal resolution. Spatial variation in strontium isotope ratios (^87^Sr/^86^Sr) is driven by the underlying geology and is independent of δ^2^H. Recently, ^87^Sr/^86^Sr has been used for the geolocation of insects and provided increased longitudinal resolution,^36^ showing that it is important to further develop technologies to advance our understanding of migratory insect population dynamics and multi-generational migratory connectivity.

Here, we investigate the spatiotemporal trans-Saharan migratory connectivity and patterns of the painted lady butterfly using δ^2^H and ^87^Sr/^86^Sr-based geographic assignment. Painted ladies are thought to cross the Sahara twice each year, with one group migrating south in the autumn and the other migrating north in the late winter and early spring.^19^^,^^21^^,^^22^^,^^23^ Through an international collaboration led by the Butterfly Migration Monitoring Scheme, a global citizen science effort, 118 painted lady samples were opportunistically collected from many sites south and north of the Sahara and the Arabian Desert, an extension of the Sahara, so that the migratory patterns along the length of the geographic barriers could be compared for both seasons. Applying dual δ^2^H and ^87^Sr/^86^Sr for continuous-surface isotope-based geographic assignment requires reliable spatial models of isotopic variation across the landscape (i.e., isoscapes). A new hydrogen isoscape was recently calibrated for butterfly wings for the Afro-Palearctic region,^37^ but a robust bioavailable strontium isoscape of this region did not exist. We compiled bioavailable ^87^Sr/^86^Sr data from the literature and completed this database with additional analysis of ^87^Sr/^86^Sr in plants from 45 sites. We used this database and applied an innovative spatial interpolation ensemble machine-learning framework to develop a strontium isoscape across the study area. Using the isoscapes, we estimated the natal origin of the collected painted lady butterflies, then estimated migration distance and direction of travel. Those isotope-based metrics allowed us to discuss the connectivity and migratory patterns of painted ladies across the Sahara and Arabian Desert.

## Results and discussion

### Strontium isotope geolocation enables new ecological insights

We developed a regional bioavailable strontium isoscape for the Afro-Palearctic region. This isoscape relies on a compilation of bioavailable ⁸⁷Sr/⁸⁶Sr data from the literature, the addition of 45 new plant ⁸⁷Sr/⁸⁶Sr measurements from Africa (*n* = 1820; Figure S1) and 14 spatial predictor variables (Table S1; Figure S2). Bioavailable strontium isoscapes are typically modeled using a random forest regression (RF) framework (e.g.,^36^^,^^38^^,^^39^). However, recent advances have demonstrated that spatial interpolation ensemble machine learning (EML) can outperform this RF framework.^40^ As the EML framework lacks direct interpretability (i.e., does not generate partial dependence plots or report the importance of predictors), we followed Le Corre et al.^40^ and selected predictors and assessed bioavailable ⁸⁷Sr/⁸⁶Sr patterns through the RF framework before using the spatial interpolation EML to produce a more accurate and unbiased strontium isoscape. We found that the spatial interpolation EML had slightly superior performance compared to RF (Figure S3). The EML isoscape is largely dominated by RF (highest absolute t-value < 2e-16) but also relies on additional base learners (i.e., gradient boosting [t-value = 0.04], support vector machines (t-value = 0.02), and generalized linear models [t-value = 0.04]).

As expected from the dominance of RF in the EML predictions, the EML isoscape showed patterns very similar to the RF-based model with ratios ranging from 0.70366 to 0.77394, with the highest ⁸⁷Sr/⁸⁶Sr in cratonic areas of Africa and the lowest ⁸⁷Sr/⁸⁶Sr along the basaltic African rift region (Figures S4A and S5A). However, the difference between spatial interpolation EML and RF-based modeling was apparent when comparing the spatially explicit uncertainty maps, with the EML isoscape more normally distributed and displaying lower average uncertainty (EML average SD = 0.00347 vs. RF average SD = 0.00448) (Figures S4B and S5B). Overall, the use of multiple learners, spatial dependencies, and an unbiased spatial cross-validation approach led to moderate performance improvements over RF by enhancing the robustness of the resulting model and the corresponding uncertainty estimates. The large range of strontium isotope ratios within Africa suggest that strontium isotopes have the potential to be a highly effective geolocation tool for the continent, especially as more bioavailable training data are produced. This regional bioavailable strontium isoscape provides a ground-breaking tool to investigate the mobility of painted lady butterflies, as well as other terrestrial insects, migratory animals (e.g., migratory megafauna, birds, early hominids^41^), archeological and modern human remains,^42^^,^^43^^,^^44^ and tissues and manufactured substrates across Africa (e.g., drugs, ivory,^45^ wood).

**Figure 1:**
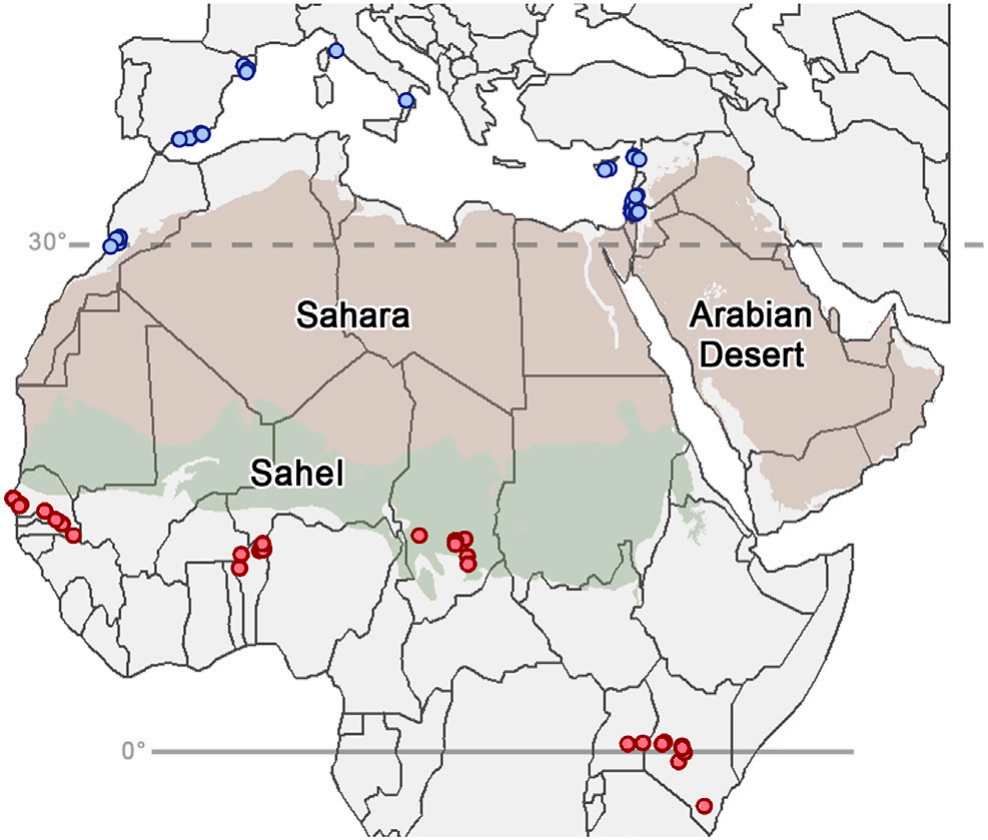
Collection sites for painted lady butterflies (*n* = 118) Butterflies were collected north of the Sahara, encompassing regions in Morocco, Spain, Cyprus, Italy, Israel, Jordan, and Syria, from January to April in 2012, 2016, 2017, and 2019 (blue circles). These butterflies represent northward-migrating painted ladies, based on our current understanding of seasonal patterns of suitable habitat.^3^^,^^19^^,^^21^ To portray southward-migrating butterflies, samples were collected south of the Sahara at multiple sites in Senegal, Benin, Chad, Uganda, and Kenya, spanning the months of August through December in 2014, 2017, 2018, and 2019 (red circles; see details in Table S2). Desert outlines were sourced from Dinerstein et al.^46^.

As a prerequisite to assessing the migratory patterns of painted lady butterflies migrating northward and southward across the Sahara, we used dual δ^2^H- and ^87^Sr/^86^Sr-based geographic assignment to estimate the natal origin of each of the collected specimens (*n* = 118; Table S2). The δ^2^H values for 69 of the individuals were previously published.^18^^,^^21^^,^^22^ Compared to the δ^2^H-based geographic assignment presented in Talavera et al.^21^ and Stefanescu et al.,^22^ δ^2^H-based estimates of natal origin in our study were broader; the highly probable area of natal origin, as estimated using a 2:1 odds ratio, was 4.4 million km^2^ larger on average. This difference in precision can be attributed to differences in isoscape uncertainty propagation between the studies. However, across all samples, the addition of strontium isotopes was able to provide additional resolution and decrease the highly probable area of natal origin by 37% on average. Our sample selection strategy was crafted to encompass migrants from both sides of the Sahara in different seasons (Figure 1); however, we were only able to confidently classify 57 of the 118 samples as having migrated (i.e., having traveled >100 km). The remaining samples had an isotopic composition (i.e., δ^2^H and ^87^Sr/^86^Sr) similar enough to their capture location that we could not discard a local origin (Figure S6). It is possible that these individuals were also migrants, but from regions of natal origin with a similar isotopic composition to their capture locations. These putative locals had isotopic compositions that are highly redundant in the Afro-Palearctic region, with ⁸⁷Sr/⁸⁶Sr ∼0.709 ± 0.001 common in most regions with marine sediments^36^ and δ^2^H typical of the warm, wet climates of the Afrotropical region.^37^ Alternatively, some of these individuals were collected later in the season and may represent the locally sourced offspring of migrants. Although the inclusion of the putative locals did not change the overall patterns detected in our study (Figure S7), we were interested in individuals that had completed their migratory movements; therefore, we took the most conservative approach and excluded these potentially local individuals from further analyses of migratory connectivity.

### Moderate migratory connectivity across the Sahara and Arabian Desert

**Figure 2:**
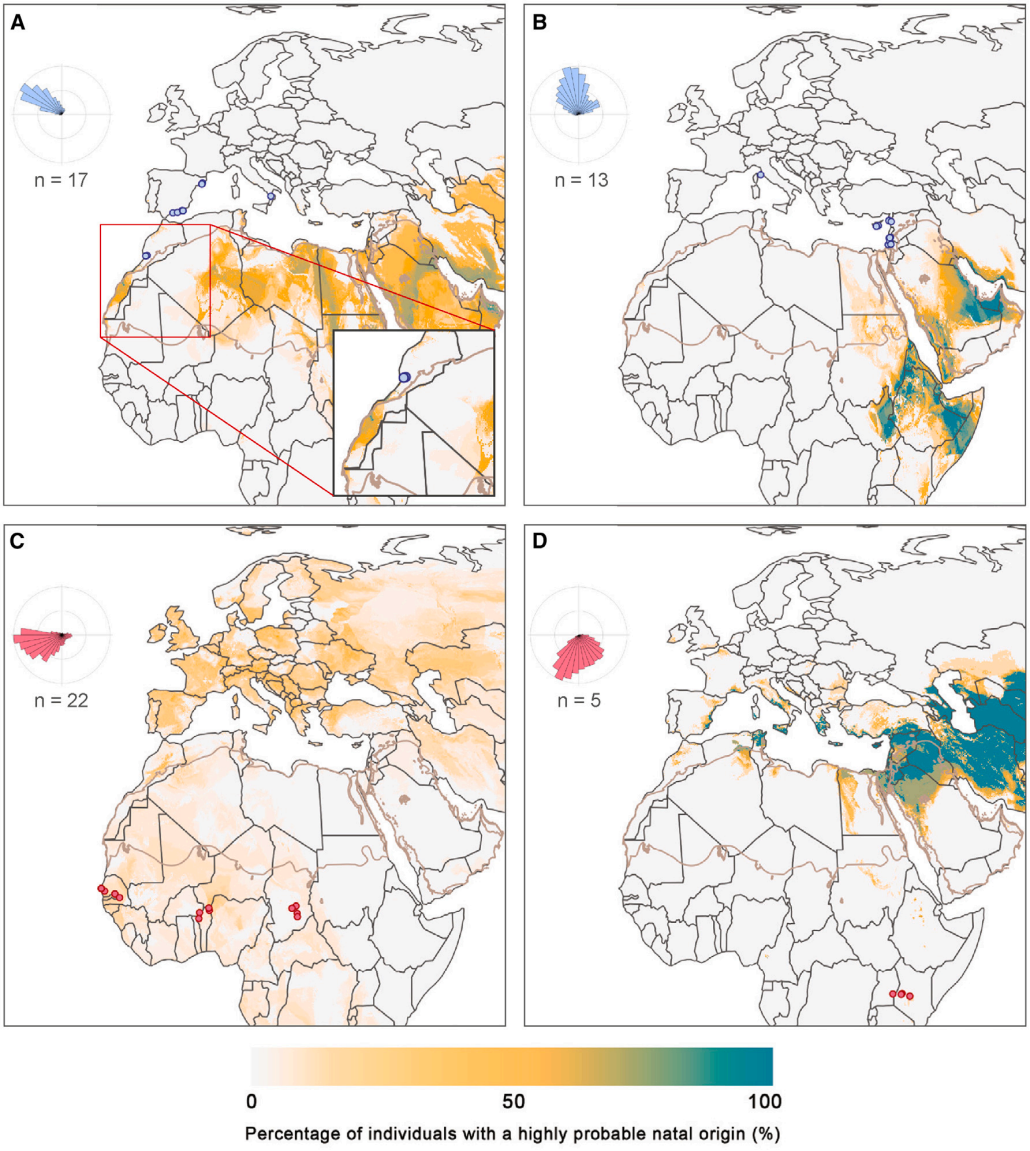
Stacked maps from the δ^2^H- and ⁸⁷Sr/⁸⁶Sr-based geographic assignment, illustrating the percentage of migratory individuals from each region with a high probable natal origin at a given location, as defined by the 2:1 odds ratio Teal areas indicate high cohesion in the estimated area of natal origin. The inset rose plots depict the combined probability-weighted estimates of the direction from the estimated natal origin (center) to the capture location. Red (south of the Sahara) and blue (north of the Sahara) circles represent butterflies that were captured from (A) northwest of the Sahara in late winter/spring (*n* = 17). The inset map highlights the high probability area of estimated natal origin in the Canary Islands and Western Sahara; (B) northeast of the Sahara in spring (*n* = 13). Included is a long-distance migrant from Italy that clustered with the northeast captures; (C) southwest of the Sahara in the autumn (*n* = 22). The rose plot is separated into samples collected from Senegal, Benin, and Chad. The rose plot for Senegal is biased toward the west because the capture locations are on the westernmost edge of the spatial extent; (D) southeast of the Sahara in late autumn (*n* = 5). See also Figures S6, S7, and S11.

As expected, rose plots of individuals captured from countries located south of the Sahara in the autumn tend to show southward bearings and rose plots of individuals captured from countries located north of the Sahara in the spring tend to show northward bearings (Figure 2). However, geographic bias can be detected in the rose plots; for example, Senegal is located at the westernmost point of the study area, and thus the rose plot is biased toward the west (Figure S8H). Individuals collected in the easternmost regions were estimated to migrate north from East Africa or the southern Arabian Peninsula in the spring (Figure 2B) and south from the northern Arabian Peninsula when collected in the autumn (Figure 2D). Although this migratory pattern has long been suspected, empirical evidence has been absent until now (e.g.,^47^). For the westernmost butterflies, there was a wider range of isotopic compositions, and high spatial redundancy of these isotopic compositions, causing the most probable regions of origin to be less cohesive than for their eastern counterparts, likely due to the broader range of sampling locations and times. During the autumn, many of the westernmost individuals showed estimates of natal origin in temperate Europe (Figures 2C and S8). Conversely, in the spring, individuals from the westernmost regions exhibited broader posterior probability surfaces, suggesting a natal origin ranging from northwestern Africa to as far east as the Arabian Peninsula (Figure 2A). Our hypothesis is that the most likely origin for the westernmost spring butterflies sampled in this study lies directly south of their capture locations in the Western Sahara and the Canary Islands (Figure 2A). This is supported by the presence of pollen from plants endemic to the Canary Islands on two of our samples captured in Spain (Sample IDs: 16C413 and 14M265).^20^ Furthermore, historical observations, recent ecological niche models, and monitoring data also suggest that the Canary Islands and areas along the coast of the Western Sahara offer highly suitable breeding conditions for painted ladies from December through to February.^19^

**Figure 3:**
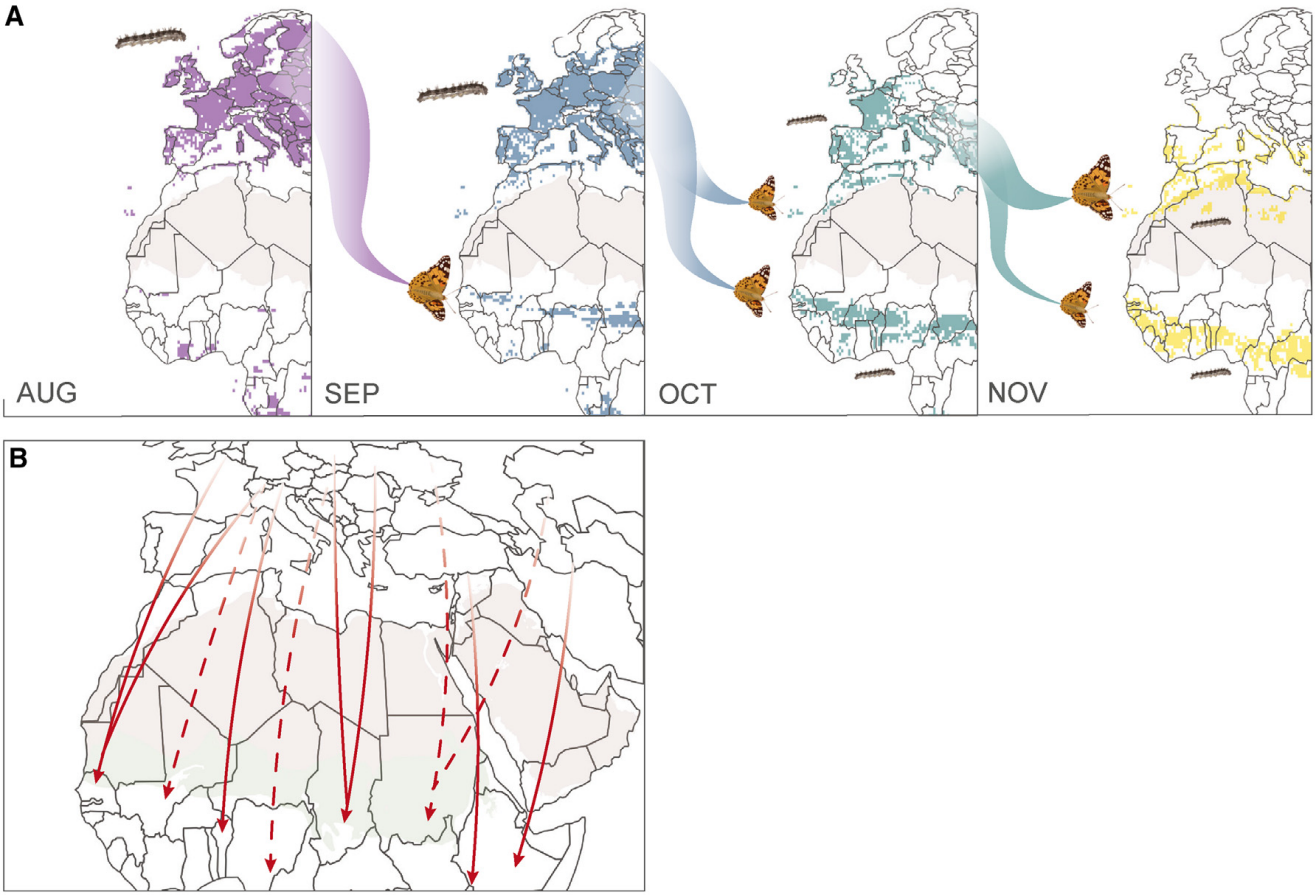
Illustrations of the proposed migratory patterns of painted lady butterflies across the Sahara (A) Conceptual model illustrating the proposed leapfrog migration pattern in the southward, autumnal segment of the annual migratory cycle in the western Afro-Palearctic. Painted lady caterpillars take between 23 and 45 days to develop into adults, and adults live for about 3–6 weeks, depending on environmental conditions.^23^^,^^26^^,^^27^^,^^28^ The maps depict monthly representations of suitable larval habitats (*P* > 95%) sourced from Talavera et al.^19^ The declining suitability of the northernmost regions over the autumn likely prompts the leapfrog migration pattern. Suitable habitat in southern destinations first appears in sub-Saharan Africa, followed by North Africa. In September, painted ladies originating from the suitable larval habitat of August (indicated in purple) migrate to sub-Saharan Africa. In October (suitable larval habitat in September indicated by blue) and November (turquoise), painted ladies from progressively southern locations migrate to either the sub-Saharan Africa or the circum-Mediterranean region.^18^^,^^19^. (B) An illustration of the hypothesized broad-front migration pattern with moderate migratory connectivity across the Sahara. Dashed lines represent our expectations should additional samples be collected between the collection sites presented in this study.

Migratory connectivity refers to the degree to which individuals from one area of the migratory range migrate exclusively to another area without mixing with other individuals from elsewhere, with strong connectivity indicating a low amount of mixing.^5^^,^^48^^,^^49^ This term is typically used to qualify the migratory patterns of birds, which often show strong connectivity and a discrete spatial structure in migratory trajectories across the Afro-Palearctic region.^5^ However, certain bird species exhibit moderate migratory connectivity, which is marked by spatial overlap in the breeding range (e.g.,^50^^,^^51^). In contrast, insects, due to their multi-generational, reticular migration patterns, typically display weaker migratory connectivity than birds.^4^^,^^5^ The distribution of painted ladies is seasonally continuous along the northern and southern sides of the Sahara^19^; a large availability of continuous habitat is sometimes correlated with stronger migratory connectivity.^13^ Indeed, the absence of large east-west movements, for example from Israel to Benin or France to Kenya, indicates spatial structure in the migratory trajectories of painted lady butterflies, pointing to moderate migratory connectivity. Moderate migratory connectivity is also supported by the moderate, statistically significant mantel correlation coefficients calculated for each season between the capture location and the centroid of the highly probable area of natal origin (spring: r_m_ = 0.40, *p* < 0.001; autumn: r_m_ = 0.22, *p* < 0.01). Thus, we propose that painted ladies show moderate migratory connectivity across the Sahara, characterized by a predominantly latitudinal, rather than longitudinal, movement pattern, suggesting parallel, broad-front migration (Figure 3B).

Strong migratory connectivity can lead to adaptation and genetic differentiation between populations.^49^ Trans-Saharan migrations in birds often show longitudinal migratory divides, where adaptive populations follow western, eastern, and, occasionally, central flyways (e.g.,^52^^,^^53^^,^^54^^,^^55^). However, the likelihood of the existence of an adaptive longitudinal migratory divide among populations of painted ladies in the Afro-Palearctic region is remote due to the complex multi-generational population dynamics of painted ladies, as is shown by the mounting evidence of inter-continental panmixia and shared demographic history.^4^^,^^18^^,^^56^ The migratory connectivity of painted ladies appears to be weaker than that observed in a few other migratory insect species, such as the fall armyworm moth *Spodoptera frugiperda* and the monarch butterfly.^5^ In these species, well-defined migration routes exist between discrete breeding grounds, separated by a geographic barrier oriented in a north-south direction (i.e., the Appalachian Mountains and Rocky Mountains, respectively).^57^ In contrast, painted ladies breed across virtually the entire longitudinal expanse of sub-Saharan Africa, with no significant geographical gaps in suitable habitat, except for tropical forests.^3^^,^^19^ We anticipate that expanding sampling efforts to cover the full extent of the Sahara would reveal a continuum of predominantly north-south movement across the desert, resulting in a broad front migration pattern (Figure 3B).

Although our data primarily support the prevalence of latitudinal migratory trajectories, we do not rule out the possibility of some longitudinal movements. A closer examination of the posterior probability surfaces for individual butterflies reveals that many individuals are probably not following perfectly north-south paths, and there may be variation in origin and migration routes even among individuals collected at the same site (see key resources table). It is likely that environmental conditions along the migration path, such as prevailing wind direction, affect the energetic costs associated with different routes and alter flight trajectories.^13^ Recent studies have shown that accidental long-distance dispersal events, often driven by longitudinal aerial highways or extreme weather events, can result in atypical movements and occurrence records (e.g.,^56^). Therefore, it is crucial to delineate migration patterns using data from many individuals to avoid overestimating the importance of irregular movements. Indisputable east-west movements were not seen for butterflies making southward, trans-Saharan journeys, whereas they were noted for some individuals captured from Europe in the spring that had estimated natal origins in North Africa or the Arabian Peninsula. An example is the individual collected in Italy, which displayed natal origins far to the east in the Arabian Peninsula (Figures 3B and S8C). This specimen was collected in April 2019 (Figure 4G), a year marked by an outbreak of painted lady butterflies attributed to anomalous vegetation growth in the Arabian Peninsula,^58^^,^^59^^,^^60^ that likely facilitated the spread of individuals from the hotspot to other parts of the range, extending as far west as Italy. Instead of indicating strong adaptation to direct north-south movement across the Sahara, the lack of east-west movements observed across the Sahara may be due to survivorship bias, as butterflies that migrate shorter distances directly across the Sahara likely experience higher survival rates, as is seen in birds.^61^ Thus, long-distance longitudinal movements between the westernmost and easternmost parts of our study area can occur but are presumably rare occurrences, particularly for trans-Saharan journeys.

**Figure 4:**
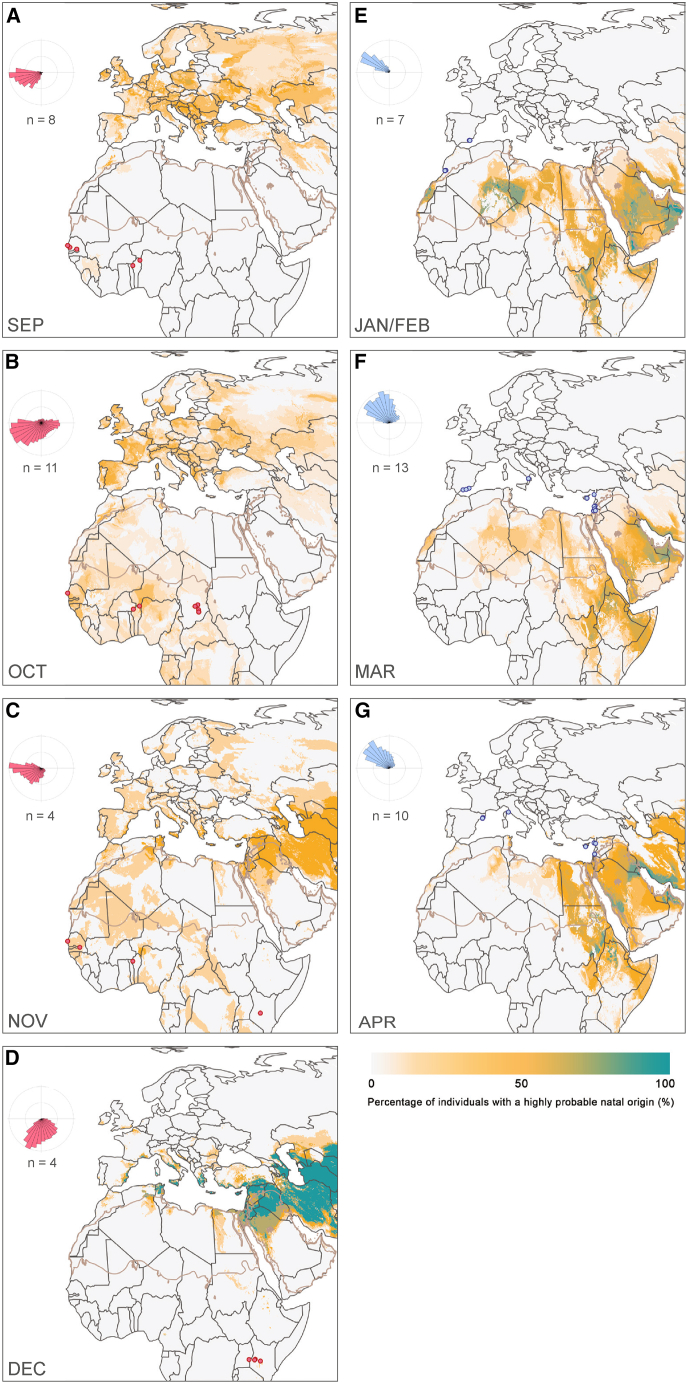
Stacked maps from the δ^2^H- and ⁸⁷Sr/⁸⁶Sr-based geographic assignment, illustrating the percentage of migratory individuals from each month with a high probable natal origin at a given location, as defined by the 2:1 odds ratio Red (south of the Sahara) and blue (north of the Sahara) circles represent individuals captured during (A) September (*n* = 8) from Benin and Senegal; (B) October from Senegal, Benin and Chad (*n* = 11); (C) November from Senegal, Benin, and Kenya (*n* = 4); (D) December captured in Uganda and Kenya (*n* = 4); (E) January (*n* = 1) and February (*n* = 6) from Spain and Morocco; (F) March from Spain, Italy, Cyprus, Israel, Jordan, and Syria (*n* = 13); and (G) April from Spain, Italy, Cyprus, Israel, and Syria (*n* = 10). See also Figure S8.

Migratory connectivity has been appraised in only a few insect species but has important conservation implications. In species with weak migratory connectivity and reticular migration patterns, local population bottlenecks can be mitigated through a compensatory demographic model.^4^ In contrast, species with stronger migratory connectivity may be more vulnerable to adverse environmental changes in specific sections of their range.^49^ The population of monarch butterflies in North America serves as a good example of this phenomenon. Although composed of a single, panmictic population, monarch butterflies show relatively strong migratory connectivity, with monarchs east of the Rocky Mountains remaining largely separate from monarchs to the west.^5^ The monarch butterflies west of the Rockies are at a high risk of local extirpation, likely due to environmental changes in that region.^62^ However, the strong migratory connectivity of the species largely prevents the larger census size east of the Rockies from bolstering the western numbers (but see^63^). Historically, the Afro-Palearctic population of painted ladies has demonstrated long-term demographic stability, albeit with large short-term population fluctuations, partially due to outbreak dynamics.^4^^,^^60^ However, in the context of global change, which can influence climate and weather events on a broad scale, even painted lady populations may be impacted. The generations of painted ladies inhabiting temperate Europe during the summer are thought to exhibit weak migratory connectivity, which may mitigate local population bottlenecks.^4^ In contrast, moderate migratory connectivity across the Sahara may reduce the compensatory abilities of the reticular migration pattern during this part of the annual cycle, leading to increased risk, particularly along the eastern and western edges of the geographic range. This is particularly pertinent during January and February when occupancy and spatial extent are at their lowest.^19^ Future studies quantifying the temporal and spatial migratory connectivity over each segment of the annual migratory cycle will be essential for a comprehensive assessment of the compensatory abilities within painted lady migration patterns and how they may be influenced by anthropogenic climate change and habitat degradation.

### Northward progression

**Figure 5:**
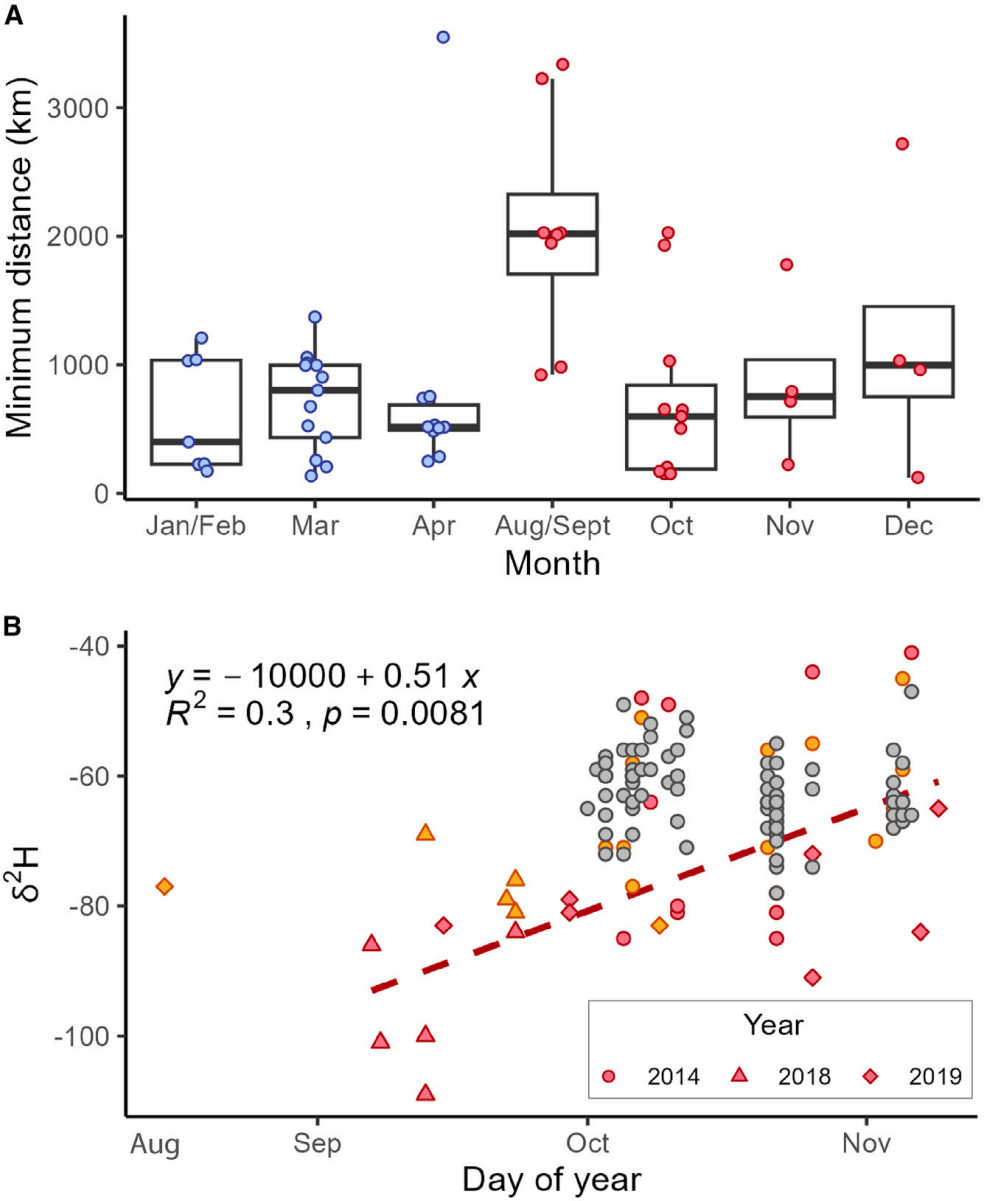
Isotope-based estimates of migratory distance and latitude of origin (A) Minimum migratory distance (km) estimates for migratory painted lady samples by month of capture (*n* = 57). The minimum distance estimate is calculated as the shortest distance from the capture location to the highly probable area of natal origin, as defined by the 2:1 odds ratio. (B) Hydrogen isotope values (‰) tend to decrease with increasing latitude. The δ^2^H values of migrants (red; *n* = 22) captured in Senegal, Benin, and Chad during the autumn have a positive relationship with the date of capture (red line; *p* = 0.008). Symbols indicate the sampling year. Putative locals are depicted in orange (*n* = 22). The positive relationship between the date of capture and δ^2^H values is supported by additional data from the literature (gray symbols; *n* = 85).^22^.

Our findings provide strong support for long-distance trans-Mediterranean and trans-Saharan migrations during the southward, autumnal segment of the annual cycle (Figures 3C, 3D, and 5A). In contrast, most of the spring migrants in our study journeyed shorter distances from northern Africa or the Arabian Peninsula into Europe (Figures 3A, 3B, and 5A). This finding reinforces the importance of North Africa as the primary source for spring migrants to Western and Central Europe^64^ and suggests that the Arabian Peninsula may be an important source for spring migrants to Eastern Europe. Talavera et al.^21^ interpreted many of the δ^2^H-based posterior probability surfaces of samples captured in Morocco and Spain as conclusively showing northward, complete trans-Saharan migrations in the spring. However, our updated dual ^87^Sr/^86^Sr- and δ^2^H-based geographic assignment discounts origins in West Africa and instead highlights the most parsimonious origins as being in the western Sahara or the Canary Islands. With the removal of conclusive isotopic evidence for northward migrations across the Sahara in the late winter and early spring, the evidence for northward movements in the west is based on species distribution models, demography, and field observations. Our sampling was insufficient to identify definitive trans-Saharan migrants or fully reconstruct northward migratory connectivity across the Sahara. We suspect that most painted ladies migrating north from sub-Saharan Africa undertake shorter journeys to breed in northern Africa or the Arabian Peninsula in late winter, with their offspring subsequently moving into Europe in a gradual northward progression. Painted ladies migrating northward across the western Sahara must do so against the prevailing northeasterly winds, known as the Harmattan, which may make journeys more energetically costly and, therefore, shorter.

Seasonal differences in the distance traveled by painted ladies show similarities to those observed in monarch butterflies. Long-distance migrations of over 3,000 km are undertaken by painted ladies in the autumn (Figure 5A). In contrast, as discussed earlier, painted ladies seem to migrate northward in the spring through several short-distance migrations. Similarly, monarchs are famous for long-distance, autumnal migrations from Canada to the overwintering grounds in Mexico, but during the spring, monarchs are known to gradually recolonize the USA and Canada in a northward progression over several generations, each covering short distances.^65^ The similarities in seasonal differences in migratory patterns between painted ladies and monarch butterflies (i.e., long-distance migrations in the autumn and a northward progression in the spring) suggest the presence of comparable environmental cues influencing the behavior of both species, such as temperature, photoperiod, and host plant development.^66^ Furthermore, the gradual northward progressions in the spring suggest an adherence to the green wave hypothesis, a phenomenon observed in migratory ungulates,^67^ bats,^68^ and birds,^69^ wherein migration closely follows seasonal vegetation growth.

### Seasonal leapfrog migration

Leapfrog migration is used to describe a migration pattern commonly found in vertebrates wherein individuals that breed further north migrate beyond other groups to locations further south. Here, we describe a probable leapfrog migration pattern for the southward, autumnal migration of painted lady butterflies in the western Afro-Palearctic. We identified that many painted ladies migrated long distances, up to over 3,500 km, from northern and central Europe to West Africa from September through to November (Figure 5A). In western Europe, δ^2^H values tend to decrease with increasing latitude.^35^ We detected an increase in the δ^2^H values of samples collected from sub-Saharan Africa over the season, suggesting a temporal, southward shift in the origin of the long-distance migrants (Figure 5B). Thus, the earliest arrivals to West Africa seem to originate from more northern latitudes. The circum-Mediterranean region is generally not a suitable breeding habitat in September,^19^ so early migrants reaching this region will be unlikely to successfully reproduce unless they continue further south to suitable breeding grounds in sub-Saharan Africa (Figure 3A). In October and November, areas in both the circum-Mediterranean region and sub-Saharan Africa contain highly suitable breeding habitat, which is progressively increasing in the circum-Mediterranean region and progressively decreasing in sub-Sahara Africa, and so painted ladies of progressively more southern origin can migrate to areas north or south of the Sahara (Figure 3A). Indeed, in October and November, painted ladies are known to migrate shorter distances from central and southern Europe to the circum-Mediterranean region.^18^ In other words, in autumn, butterflies bred in northern Europe migrate further south than many butterflies bred in southern Europe, suggesting a leapfrog migration pattern in the western Afro-Palearctic. In winter, painted ladies in the Afro-Palearctic breed in two separate areas: the circum-Mediterranean region north of the Sahara and tropical Africa close to the equator.^3^^,^^19^ Monitoring data suggest that most offspring of the leapfrogging individuals will remain within their respective regions to breed during the winter.^19^^,^^27^ To confirm this leapfrog migration pattern, future technological advances in lightweight biologgers and their subsequent large-scale deployment will be necessary.

Isotope-based geographic assignment has helped identify leapfrog migration patterns in birds (e.g.,^70^^,^^71^^,^^72^). Additionally, similar temporal shifts in δ^2^H were recently detected in *Danaus gilippus* and attributed to a leapfrog migration pattern, although a short-distance migratory group was not identified in the study.^73^ However, the leapfrog migration in painted ladies we propose here differs from the classic vertebrate leapfrog migration pattern in several ways. Leapfrog migrations in birds are generally performed by two, often genetically differentiated, populations. The spatial segregation between the two populations is thought to be driven primarily by competition, pointing to an evolutionary process behind the migration pattern.^74^ In contrast, painted ladies in the western Palearctic/Afrotropical region are panmictic,^18^ pointing to behavioral plasticity as the main mechanism by which painted ladies from northern origins migrate further south. Thus, the leapfrog migration pattern noted in the autumnal segment of the annual migratory cycle is likely driven by ecological processes. Environmental cues in northern Europe (e.g., Scandinavia), such as photoperiod, temperature, and host plant phenology, change earlier in the late summer and autumn (i.e., September) and could prompt migratory behavior.^66^^,^^75^^,^^76^ Although there are expected to be additional costs associated with the long-distance migration from northern Europe to sub-Saharan Africa, such as decreased survival during migration,^77^ these drawbacks may be outweighed by the advantages of being early arrivals to the region, as has been proposed in the context of birds.^78^ This advantageous position may bring benefits such as a release from parasites, access to a high abundance of larval host plants, and reduced competition.

### Migratory patterns in the eastern Sahara

The broad spatiotemporal coverage of our sampling on the western side of the range, particularly for the early autumn, facilitated the identification of a leapfrog migration. In contrast, we only have samples from November and December for the easternmost samples, limiting our ability to identify early autumn long-distance movements in that part of the range (Figures 4C and 4D). Additional studies with higher spatiotemporal sampling in central and eastern Africa, as well as in the Arabian Peninsula, will be required to detect potential northern Palearctic origins and test the possibility of a leapfrog migration in the east. However, we were able to detect long-distance migrations from the Eastern Mediterranean to Kenya in November and December, providing the first empirical evidence of direct migration from the eastern Palearctic to eastern tropical Africa (Figures 4C and 4D). The distribution of painted ladies in the Palearctic region extends from Portugal to Japan. The migratory patterns of the easternmost butterflies in our study highlight the role of the Arabian Peninsula as a crucial stepping-stone connecting Europe, Africa, and Asia (Figures 3B and 3D).^79^^,^^80^ Connections between East Africa and Asia have been proposed for other migratory insects, such as the globe skimmer *Pantala flavescens*.^81^^,^^82^ Subsequent research should delineate the migratory patterns of painted lady butterflies across the entire Palearctic and quantify the extent of east-west connections between Europe/Africa and Asia, which could be partially achieved by examining east-west patterns of genetic isolation by geographic distance.

### Future work

Although we have begun to characterize the broad-scale migration patterns of painted lady butterflies across the Sahara, many other questions regarding the ecology and behavioral adaptations of insects navigating this challenging terrain persist. These questions include the following: what environmental cues prompt insects to either embark on desert crossings or halt their migration, what is the survival rate of desert crossings, and how do insects adjust their physiology, flight altitude, or diurnal migration timing to successfully traverse the Sahara. Many trans-Saharan migratory birds have been observed changing their behavior while crossing the desert (e.g.,^53^), and it is possible that insects do the same. For example, numerous songbirds switch from migrating during the day to migrating at night and resting in the shade of sand dunes or rocks during the day.^83^ Painted ladies are able to stay airborne for several days, as evidenced by their accidental crossings of the Atlantic Ocean.^56^ However, a knowledge gap remains concerning the fuel usage and recharge strategies of painted ladies. Little is known about the extent to which they exploit floral and water resources in oases, wadis, and mountain ranges, such as the Ahaggar and Tibesti Mountains. The impact of migratory painted ladies and other insects on the delicate ecosystems of desert regions, the Sahel, and the sub-Saharan savanna is virtually unknown. Given anthropogenic climate change and widespread habitat degradation, delineating insect migratory patterns and understanding the ecological importance of migratory insects in the Sahara are important for ensuring the preservation of critical ecosystem services.

### Limitations of the study

Although isotope-based estimates of natal origin allowed us to explore the trans-Saharan migratory patterns and migratory connectivity of painted lady butterflies in remarkable detail, some caveats remain. The combination of δ^2^H and ^87^Sr/^86^Sr for geographic assignment provided more specific estimates of natal origin than using a single isotope. However, some individuals exhibited particularly broad estimated areas of natal origin (4 million km^2^ on average), often from fragmented and distant areas, due to the redundancy of certain isotopic compositions across the landscape. These nonspecific estimates of natal origin can potentially introduce inaccuracies in downstream metrics, such as estimates of migration distance.^18^^,^^84^ In order to overcome this limitation, we mainly interpreted our results at the population level by overlaying the posterior probability surfaces of multiple individuals and presenting a conservative estimate of migration distance (i.e., minimum distance) for many individuals. Future studies could combine δ^2^H and ^87^Sr/^86^Sr geolocation with additional isotopes, such as sulfur isotopes, or other geolocation techniques, like wind trajectory analysis or pollen metabarcoding, to further refine the estimates of natal origin (e.g.,^56^). Additionally, in contrast to the findings of Talavera et al.,^21^ which relied solely on δ^2^H, our approach combining δ^2^H and ^87^Sr/^86^Sr was unable to detect definitive trans-Saharan migrants northwest of the Sahara. Nevertheless, northward movements from sub-Saharan Africa are likely to occur, given the larger spatial extent of painted lady breeding areas present in sub-Saharan Africa, compared to North Africa, from December to February.^19^ To complete the northward migration and connectivity model of painted ladies across the Sahara, future research should concentrate on sampling during late winter (e.g., February) and in locations closer to the northern edge of the Sahara (e.g., Tunisia, Libya), or in the Sahara, to capture complete northward trans-Saharan migrants.

## Resource availability

### Lead contact

Further information and requests for resources should be directed to and will be fulfilled by the lead contact, Megan S. Reich (mreic084@uottawa.ca; meganreich13@gmail.com).

### Materials availability

This study did not generate new unique reagents.

### Data and code availability

• Data: all data generated from this study are deposited with the Open Science Framework and publicly available as of the date of publication.
• Code: all R codes used in this study are deposited with the Open Science Framework and publicly available as of the date of publication.
• Any additional information required to reanalyze the data reported in this paper is available from the lead contact upon request.

## Acknowledgments

Special thanks go to Kerry Klassen and Paul Middlestead at the Ján Veizer Stable Isotope Laboratory, Kathy Gordon at the Pacific Center for Isotopic and Geochemical Research, Alexandre Voinot at Queen’s Facility for Isotope Research, Shuanquan Zhang at Carleton University, and André Poirier at the GEOTOP-UQAM for their expert assistance with the isotopic analyses. We also thank the many collaborators and community scientists of the Butterfly Migration Monitoring Scheme (www.butterflymigration.org) who collected samples, including A. Aristophanous, F. Bahleman, L. Dapporto, M. Gascoigne-Pees, D. Glazner, E. Goudegnon, R. Hilal, K. Kebé, M. Kiki, S. Lifshitz, M. Menchetti, T. Oron, A. Orteu, E. Plana, M. Salimeh, S. Scalercio, S. Schär, T. Suchan, O. Tomer, E. Toro-Delgado, and I. Tzuk-Kovachy. This study was funded by grant 2018-00738 of the New Frontiers in Research Fund (Government of Canada) to G.T. and C.B.; the NSERC Discovery Grant RGPIN-2019-05709 to C.B.; and by the 10.13039/100006363National Geographic Society (grant WW1-300R-18), the 10.13039/501100000409British Ecological Society (grant LRB16/1015), the grant LINKA20399 from the CSIC iLink program, the grant PID2020-117739GA-I00 and PID2023-152239NB-I00 (MCIN/AEI/10.13039/501100011033), and the grant 2021-SGR-01334 (Departament de Recerca i Universitats, 10.13039/501100002809Generalitat de Catalunya) to G.T. M.R. was supported by the Queen Elizabeth II Graduate Scholarship in Science and Technology (QEII-GSST), the Ontario Graduate Scholarship, and the 2021 ORIGIN project (NSF award DBI-1565128).

## Author contributions

G.T. and C.B. conceived the study; G.T. and R.V. coordinated the sample collection; M.R., I.N., D.B., R.V., and G.T. collected the samples; M.R., S.G., L.H., S.Z., and C.B. performed the isotopic analyses; S.G., C.B., and M.L. modeled the strontium isoscape; M.R. performed the data analysis; M.R. and S.G. led the writing of the manuscript; all authors contributed to the drafts and gave final approval for publication.

## Declaration of interests

The authors declare no competing interests.

## STAR★Methods

### Key resources table

**Table:** 

REAGENT or RESOURCE	SOURCE	IDENTIFIER
**Deposited data**		

Sample metadata, isotope data, code, and posterior probability surfaces	This paper	Open Science Framework: https://doi.org/10.17605/OSF.IO/6VNY3
Painted lady hydrogen and strontium isotope data	Reich et al.^18^	GTcoll18B773, GTcoll18B774, GTcoll18B775, GTcoll18B782, GTcoll19H128, GTcoll19H129, GTcoll19H137, GTcoll19H140, GTcoll18B699, GTcoll18B705, GTcoll18B720, GTcoll18B721, GTcoll18B722, GTcoll19H115, GTcoll19H116, GTcoll19H119, GTcoll19H120, and GTcoll19H121
Painted lady hydrogen isotope data	Stefanescu et al.^22^	RVcoll14T597, RVcoll14T630, RVcoll14T645, RVcoll14T653, RVcoll14T867, RVcoll14T876, RVcoll14T715, RVcoll14T075, RVcoll14T186, RVcoll14T192, RVcoll14T202, RVcoll14T240, RVcoll14T273, RVcoll14T280, RVcoll14T305, RVcoll14T339, RVcoll14T366, RVcoll14T378, RVcoll14T967, RVcoll14U004, RVcoll14U014, RVcoll14U016, RVcoll14U018, RVcoll14U050, RVcoll14U061, and RVcoll14U074
Painted lady hydrogen isotope data	Talavera et al.^21^	RVcoll17A040, RVcoll17A094, RVcoll17A114, RVcoll17A139, RVcoll17A157, RVcoll17A159, RVcoll17A162, RVcoll17A167, RVcoll17A173, RVcoll17A174, RVcoll17A175, RVcoll17A179, RVcoll17A184, RVcoll17A187, RVcoll17A209, RVcoll14M265, RVcoll14M274, RVcoll14M281, RVcoll14M282, RVcoll16C403, RVcoll16C407, RVcoll16C409, RVcoll16C413, RVcoll16C416, and RVcoll16C425
Monthly maps of suitable larval habitat	Talavera et al.^19^	N/A

**Software and algorithms**		

R v4.3.2	R Core Team^85^	https://www.r-project.org/

### Experimental model and study participant details

#### Painted lady sample collection

The multi-generational annual migratory cycle of the painted lady butterfly is characterised by spatiotemporally overlapping generations, and thus trans-Saharan migrants do not necessarily migrate at the same time; instead, painted ladies arrive on the far side of the Sahara over a period of weeks or months, the local-scale destinations of which vary between years^4^^,^^60^. The Butterfly Migration Monitoring Scheme, a global citizen science effort (butterflymigration.org), monitors and collects painted lady butterflies and archives the samples in a scientific collection hosted at the Institut Botànic de Barcelona and the Institut de Biologia Evolutiva. Samples are processed upon arrival at the collection by separating wings from bodies and storing the wings in glassine envelopes in a dry location at room temperature. From this collection, some samples have had hydrogen isotopes analyzed as part of previous studies^21^^,^^22^; from this subset of samples, we chose to analyze strontium isotope ratios for individuals whose hydrogen isotope values indicated migratory movement. We chose additional samples from the collection to give the best possible coverage of the length of the Sahara, both north and south of the Sahara. The collection did not yield synchronously collected samples, so samples from a range of dates were chosen to give us the best chance of selecting trans-Saharan migrants. In total, 118 painted ladies were included in this study (Figure 1 and Table S2). The effect of sex on migratory patterns was not assessed, but previous studies have found no sex-based differences in the migratory behavior of painted ladies.^18^

### Method details

#### Hydrogen isotope analysis

Of the 118 samples, the δ^2^H of 69 samples have already been reported in the literature.^18^^,^^21^^,^^22^ However, some of these samples were analyzed using older standard δ^2^H values (ORX: −35.4‰, DS: −172.7‰ and KHS: −54.1‰, CBS: −197‰, respectively). To ensure that these measurements were compatible with the hydrogen isoscape^37^ and with the other samples analyzed in this study, the δ^2^H values were converted back to the international standard scale, Vienna Standard Mean Ocean Water - Standard Light Antarctic Precipitation (VSMOW-SLAP), using the *reftrans* function in the *assignR* package in R.^86^^,^^87^

The non-exchangeable δ^2^H of the remaining 49 samples were measured at the Ján Veizer Stable Isotope Laboratory at the University of Ottawa, Canada. Prior to δ^2^H analysis, a forewing from each butterfly was soaked, with agitation, in three successive baths (1 h, 30 min, 10 min) of 2:1 chloroform:methanol solution to remove surficial dust and lipids, which are known to introduce error into δ^2^H measurements,^88^^,^^89^ then dried in the laboratory oven at 50°C for over 24 h. Samples were carefully cut from the wing to reduce intra-individual variation from differing pigmentation^88^ and the presence of wing veins.^32^ Samples were then weighed (0.150 ± 0.010 mg) into silver capsules and loaded into a zero-blank autosampler (Thermo, Germany). All measurements were taken using high temperature (1,400°C) flash pyrolysis (TCEA, Thermo Finnigan, Germany) with a helium carrier passed through a chromium-filled reactor and, after separation, introduced via a Conflow IV interface (Thermo Finnigan, Germany) into a Delta V Plus IRMS (Thermo Finnigan, Germany).

Two different analytical methods were used. The first 41 samples were subjected to the equilibration with dual waters approach, following the methodology outlined in Meier-Augenstein et al.^90^ Briefly, two aliquots of each wing sample and isotope standards were weighed into silver capsules One aliquot was placed in a dessicator with isotopically “light” water (−398 ± 2.0‰) and the other was placed in a dessicator with isotopically “heavy” water (15.6 ± 2.0‰) for 4 days to equilibrate with their respective waters. Then the capsules were placed under vacuum for at least 7 days at room temperature (20°C) to dry. Samples were then loaded onto a Pyrolysis Elemental Analyser with a zero-blank autosampler (TC/EA, Thermo, Germany) interfaced with a ConFlo IV (Thermo, Germany) to an IRMS (DeltaPlus XP, Thermo, Germany). The δ^2^H values were normalized to two reference materials with non-exchangeable H: IAEA-CH-7 (−100.3‰), and an in-house kaolinite (−58.0‰). The production of HCN was not accounted for.^91^ All reported δ^2^H values are reported to the international scale VSMOW-SLAP. Analytical precision for δ^2^H was ±2.0‰.

The non-exchangeable δ^2^H of the final 8 samples were determined using the comparative equilibrium approach,^92^ as in Bataille et al.^93^ To maintain uniformity and ensure consistency across these analytical protocols, we selected 20 insect samples for duplicate analysis using both protocols (i.e., comparative equilibrium and equilibration with dual waters). Through this comparative analysis, a calibration equation was developed between the two methods (Figure S9) and used to ensure all δ^2^H values were on the same scale. All new sample δ^2^H are reported based on a three-point calibration using: CBS (caribou hoof; −157 ± 0.9‰^94^), KHS (kudu horn; −35.3 ± 1.1‰^94^), and USGS43 (human hair; −44.4 ± 2.0‰^95^). To assess the quality of the measurements, one keratin reference standard, USGS42 (human hair; measured: −75.3 ± 0.5‰, *n* = 4; standard: −72.9 ± 2.2‰^95^), as well as two in-house chitin standards, ground and homogenised *Lymantria dispar* (measured: −64.4 ± 1.8‰, *n* = 6; long-term average: −64 ± 0.8‰) and Alfa Aesar chitin (measured: −22.8 ± 0.7‰, *n* = 4; long-term average: −22 ± 1.2‰), were measured as internal standards. Based on within-run replicates of the internal standards and repeated sample measurements, the precision of all measurements is estimated to be about ±2‰. All reported δ^2^H values are normalised to the VSMOW-SLAP standard scale.

#### Strontium isotope analysis

Of the 118 samples, the ^87^Sr/^86^Sr of 18 samples have already been reported in the literature.^18^ The ^87^Sr/^86^Sr analyses for the remaining samples were performed in three analytical batches at different facilities. To prepare the first batch of 25 samples, a single forewing was washed in a 2:1 v/v chloroform:methanol solution in two successive washes. This solution-based cleaning protocol removes surficial dust similarly to cleaning with pressurized nitrogen gas^36^ but has a higher risk of Sr contamination.^33^ Therefore, the nitrogen gas protocol was chosen for future batches. Three of these samples were digested in 4 mL of 16 M HNO_3_ using a MARS 6 microwave digestion system (CEM Corporation, USA). The rest of the samples were digested at 100°C for 48 h on a hot plate using 1 mL 16 M HNO_3_. All samples had 1 mL 10 M H_2_O_2_ added to complete digestion. Sample preparation was performed in a fume hood providing class 100 conditions. The separation of strontium was performed in microcolumns loaded with 125 μL of Sr-spec Resin (Eichrom Technologies, LLC). The matrix was rinsed out twice using 800 μL 7 M HNO_3_ and Sr was collected with two passes of 800 μL H_2_O. The first batch was measured at the Queen’s Facility for Isotope Research at Queen’s University, Canada, in January 2019 using a Neptune multi-collector inductively coupled plasma mass spectrometer (MC-ICP-MS; ThermoScientific, Bremen, Germany) coupled to a Micro-FAST syringe injection system. The reproducibility of the ^87^Sr/^86^Sr measurement was 0.71020 ± 0.00006 (1 SD, *n* = 29) for 1 ppb NIST SRM987.

To prepare the second and third batch for ^87^Sr/^86^Sr analysis, a single forewing from each butterfly was cleaned using pressurized nitrogen gas for 2 min at 69 kPa to remove any surface contaminants (e.g., dust).^36^ The wings were then digested using microwave digestion (Anton Paar Multiwave 7000; Austria) in 1 mL HNO_3_ (16M; distilled TraceMetal Grade; Fisher Chemical, Canada). The separation of Sr was processed using a protocol described in Hu et al.^96^ The remaining steps, including temperature adjustment and duration, aliquot preparation, and analysis for Sr content via inductively coupled plasma mass spectrometry (ICP-MS; Agilent 8800 ICP-QQQ, Agilent Technologies Inc., CA, USA), were consistent with the procedures outlined in Reich et al.^33^ Calibration standards were prepared using single-element certified standards obtained from SCP Science (Montreal, Canada). After separation, eluates were dried and re-dissolved in 200 μL 2% v/v HNO_3_ for ^87^Sr/^86^Sr analysis. The second batch (*n* = 16) was measured at GEOTOP-UQAM in Montreal, Canada in July 2021 with a Nu-Plasma II MC-ICP-MS (Nu Instruments) coupled to a desolvating nebulizer (Aridus II, CETAC Technologies). The reproducibility of the ^87^Sr/^86^Sr measurement was 0.71022 ± 0.00006 (*n* = 6) for 5 ppb NIST SRM987 and 0.71022 ± 0.00002 (*n* = 14) for 25 ppb NIST SRM987. The reproducibility of an in-house pure Sr standard was 0.70815 ± 0.00002 (*n* = 4). The third batch (*n* = 59 plus the 18 samples presented in Reich et al.^18^) was measured at the Pacific Center for Isotopic and Geochemical Research at the University of British Columbia, Canada, in December 2021 using a Nu-Plasma II MC-ICP-MS (Nu Instruments) coupled to a desolvating nebulizer (Aridus II, CETAC Technologies). The reproducibility of the ^87^Sr/^86^Sr measurement for 5 ppb NIST SRM987 was 0.71025 ± 0.00009 (*n* = 138) and 0.71019 ± 0.00011 (*n* = 48) for 1.4 ppb NIST SRM987. A matrix-matched chitin internal standard, 5 ppb Alfa Aesar chitin, was also used (0.713959 ± 0.00009; *n* = 3).

Instrumental mass fractionation was corrected by normalising ^86^Sr/^88^Sr to 0.1194 using the exponential law.^97^ The isotopes ^84^Sr, ^86^Sr, and ^87^Sr have isobaric interferences from ^84^Kr, ^86^Kr, and ^87^Rb, respectively, and, in all cases, were corrected for using the ^85^Rb and ^83^Kr signals. Procedural blanks contained 200 pg of strontium, on average (maximum = 335 pg). The uncertainty introduced by the preparation procedure was quantified as the product of the uncertainty of the ^87^Sr/^86^Sr measurement of a representative procedural blank and the ratio between the signal intensity of the blank and the sample.^98^ This uncertainty was then propagated with the measurement uncertainty and is reported in Table S2; for samples with low strontium mass, the bias introduced by procedural blanks contributed substantially to the uncertainty (maximum = 50% increase).

#### Bioavailable strontium isoscape for the Afro-Palearctic

##### Strontium isotope compilation for the Afro-Palearctic region

We compiled 8,755 bioavailable ⁸⁷Sr/⁸⁶Sr measurements from Europe, the Middle East, and Africa from 308 literature studies (see key resources table). Our literature compilation encompasses 35 more studies specific to the Afro-Palearctic range compared to Bataille et al.^38^ In the majority of cases, geographic coordinates were reported by the authors in the publication. When geographic coordinates were not included, we used Google Earth to georeference geographic information for each sample by using locality names and maps. When needed, authors were contacted for clarification purposes regarding sample locality. We converted all data to decimal degrees for consistency.

We contributed an additional 45 plant ⁸⁷Sr/⁸⁶Sr measurements from 12 countries across Africa, Europe, and the Middle East to the literature compilation. Using the compilation from the literature, we identified critical spatial data gaps and filled them by procuring plant samples from the collection of the Institut Botànic de Barcelona (IBB, CSIC) with precise georeferencing and metadata (Figure S1). Plant samples were selected to maximise geological variability over the study area. The selected plants are from various taxa (mostly the Asteraceae family with some Malvaceae and Urticaceae) and were sampled from 12 countries in Africa between 2014 and 2021. We subsampled ∼1 g of plant tissue per sample location, pooling 2 to 5 plants for each location. We cleaned the plants to remove all surface mineral dust by placing the cut sample in distilled deionized (DDI) water in the ultrasonic bath for 15 min. The plants were then rinsed another 2 times with DDI water and with ultrapure deionized H_2_O (18.2 MΩ cm @ 25°C). Plant samples were then dried in the oven at 70°C. We digested the plant samples using microwave digestion, analyzed their Sr concentrations, isolated their Sr, and determined their ⁸⁷Sr/⁸⁶Sr using preparation protocols and analytical approaches identical to those described for the painted lady butterfly samples. All our plant samples underwent ⁸⁷Sr/⁸⁶Sr analysis at the Pacific Center for Isotopic and Geochemical Research at the University of British Columbia.

Altogether, the compiled dataset greatly improves the number of samples available for Africa, relative to Bataille et al.^38^ (*n* = 1820 vs. *n* = 939). Although the coverage is greatly improved, the sampling density still varies across the study area with a higher density of bioavailable ⁸⁷Sr/⁸⁶Sr data in Europe (Figure S1). The dataset displays a wide range of ⁸⁷Sr/⁸⁶Sr spanning from 0.7025 to 0.8264 with bioavailable ⁸⁷Sr/⁸⁶Sr ranging between 0.7040 and 0.7885 in Africa.

##### Auxiliary variables for the regression models

We assembled a catalog of 28 environmental and climatic geospatial data known to influence bioavailable ⁸⁷Sr/⁸⁶Sr variation according to Capo et al.^99^ and Bataille et al.^38^ (Table S1). We used the same ensemble of variables as in Bataille et al.^38^ to represent geology, soil properties, relief, climate and agricultural activity and introduced new atmospheric deposition variables, including volcanic ash,^100^ aerosols,^101^ and anthropogenic deposition.^99^ We reprojected and resampled each of these variables into WGS84-Eckert IV at 1 km resolution. Using the sample locations from our ⁸⁷Sr/⁸⁶Sr metadata compilation, we extracted the local pixel values for each of these 28 tested predictors.

We used the *VSURF* package (Variable Selection Using Random Forest) to optimise and identify the most important predictors of bioavailable ⁸⁷Sr/⁸⁶Sr from the 28 variables.^102^ Fourteen dominant variables were identified to predict bioavailable ⁸⁷Sr/⁸⁶Sr across the Afro-Palearctic range (Figure S3A). These predictors are similar to those selected in previous efforts to map bioavailable ⁸⁷Sr/⁸⁶Sr in other study areas (e.g.,^36^^,^^38^^,^^39^^,^^103^^,^^104^). As in many other regions, geological variables, including the age of the underlying terranes or geological units and the predicted bedrock ⁸⁷Sr/⁸⁶Sr, dominate, reflecting the influence of age and lithology,^105^ with younger and more mafic geological units transmitting lower ratios to ecosystems than older, more felsic, units (Figures S2B and S2I). Soil properties, notably pH and clay content, show a strong relationship with bioavailable ⁸⁷Sr/⁸⁶Sr, with higher soil pH and lower clay content usually correspond with lower bioavailable ⁸⁷Sr/⁸⁶Sr because more alkaline soils often reflect carbonate-dominated underlying bedrock with lower ⁸⁷Sr/⁸⁶Sr (Figures S2F and S2H).^99^ As expected for this desert-dominated region, the deposition of dust aerosols is an important contributor to bioavailable ⁸⁷Sr/⁸⁶Sr with higher dust inputs corresponding to high bioavailable ⁸⁷Sr/⁸⁶Sr (Figure S2G).^38^^,^^104^ Higher sea salt aerosol deposition leads bioavailable ⁸⁷Sr/⁸⁶Sr to converge toward the seawater ratios of 0.7092 (Figure S2C).^38^ Interestingly, several of the newly incorporated atmospheric deposition variables were selected as important predictors of bioavailable ⁸⁷Sr/⁸⁶Sr and reflect the key inputs to soils of black carbon (Figure S2D)^99^^,^^106^ and volcanic ash (Figure S2A).^100^

##### Machine-learning regressions

We first predicted bioavailable ⁸⁷Sr/⁸⁶Sr across the Afro-Palearctic region using random forest regression (RF) through the *caret* package^107^ using the framework of Bataille et al.^38^ This framework used the bioavailable ⁸⁷Sr/⁸⁶Sr compilation and the 14 dominant predictors identified by *VSURF* to predict bioavailable ⁸⁷Sr/⁸⁶Sr across the Afro-Palearctic. One of the known issues with RF is the absence of consideration of spatial dependence structure in the data. Several approaches have been proposed to solve this issue including adding “geographical features,” such as buffer or oblique distances,^108^ or combining multi-scale RF models.^109^ Bataille et al.^38^ demonstrated how RF produces overly confident predictions and extrapolation in data-poor regions.

To address some of these challenges, we use an innovative framework for modeling bioavailable ⁸⁷Sr/⁸⁶Sr by applying a spatial interpolation ensemble machine-learning regression (EML) approach through the *landmap* package.^40^^,^^110^ The *landmap* framework accounts for spatial dependency by incorporating buffer distances around each point as covariates in the regression while keeping computational needs limited by using oblique distances and PCA to summarise those spatial dependencies. To further account for spatial dependency and geographic sampling biases, the modeling framework applies a spatial cross-validation approach. Additionally, EML combines a series of machine-learning algorithms, or learners, through the use of a meta learner. This combination improves the performance and robustness of the model by limiting the biases of using a single algorithm (e.g., RF). Following Le Corre et al.,^40^ we used the training dataset and the predictors selected by the RF regression analysis to apply the spatial interpolation EML. We used the default setting to make predictions where the meta-learner is generated from the linear model from five independently-fitted learners including (i) RF, (ii) optimised distributed gradient boosting, (iii) support vector machines, (iv) neural network, and (v) lasso and elastic-net regularized generalised linear models. The *landmap* framework generates mean prediction maps, which represent the best estimate of bioavailable ⁸⁷Sr/⁸⁶Sr, as well as prediction errors. These prediction errors were obtained through quantile forest regression, implemented in *landmap* via the *forestError* package,^111^ and estimated from the lower and upper 67% quantile (∼1 standard deviation on the Gaussian distribution). To ensure that the uncertainty estimates were conservative, we added an estimate of intra-site variation (i.e., 0.001) to the prediction errors.

The n-fold cross validation of the spatial interpolation EML demonstrated slightly superior performance to the RF framework (RSME = 0.0029; R^2^ = 0.74 vs. RMSE = 0.0031; R^2^ = 0.73; Figures S3B and S3C). The EML isoscape algorithm incorporates spatial dependencies and uses an unbiased spatial cross-validation approach leading to more robust uncertainty estimates.^40^^,^^110^ However, this approach is best applied for local to regional bioavailable ⁸⁷Sr/⁸⁶Sr predictions as computational time becomes prohibitive for large datasets.^40^^,^^110^

### Quantification and statistical analysis

#### Isotope-based geographic assignment

The natal origin of each of the 118 painted lady samples was estimated using continuous-surface isotope-based geographic assignment via the *assignR* package^87^ in R v4.3.2.^85^ First, a growing-season amount-weighted precipitation isoscape, sourced from waterisotopes.org, using the OIPC v3.2 from Bowen et al.,^35^ was calibrated to butterfly wing tissue using the linear relationship between precipitation and a calibration dataset of residential butterflies from across Europe and Africa,^37^ resulting in a wing chitin hydrogen isoscape (δ^2^H_wing_ = −39.80 + 0.80 x δ^2^H_GSP_, r^2^ = 0.53). The hydrogen isoscape was resampled to match the resolution and extent of the strontium isoscape. The isotopic composition of each unknown-origin painted lady was then compared, using a normal probability density function, to the butterfly wing tissue hydrogen isoscape and the aforementioned bioavailable strontium isoscape to estimate the probability that each pixel of the isoscapes was the natal origin.

The resulting posterior probability surfaces were summarised into binary surfaces using the 2:1 odds ratio (0.3 isopleth), wherein the top third of the probability distribution was re-coded as highly probable (i.e., 1) and the remaining pixels were re-coded as low probability (i.e., 0). A conservative estimate of migration distance, minimum distance, was measured as the shortest distance from the capture location to the highly probable area of natal origin (Figure S10).^18^^,^^84^^,^^112^ Next, we screened for “putative locals” butterflies that had a high probability of originating from their capture location or had a minimum distance of less than 100 km. These butterflies are either (1) local or regional butterflies that have developed in the capture area, or (2) migrants from a far-off location that has the same isotopic composition as the capture. Although the inclusion of the putative locals did not change the overall patterns detected in our analysis (Figure S7), we conservatively assessed migratory connectivity using only 57 unambiguous migrants. Binary surfaces were summed and then normalised by the number of individuals, resulting in “stacked maps” representing the percentage of individuals with a high probability of originating from each pixel. Stacked maps were also created by grouping by region of capture (Figure 2), month of capture (Figure 4), and country of capture (Figure S8). Migrant butterflies from north and south of the Sahara were also separately grouped by their isotopic composition using centroid clustering (Figure S11). A modification of the *wDist* function of the *assignR* package was used to estimate the direction that was traveled using a distance-weighted probability density function.

Finally, migratory connectivity was quantitively assessed for each season using mantel correlation coefficients by testing for a correlation between the matrix of distances of the capture locations and the centroids of the highly probable natal origin (i.e., the binary surface; Figure S10). A strong correlation is expected if the distances between the capture locations of painted ladies match the distances between their natal origins, and would indicate strong migratory connectivity.^113^ The quantification of migratory connectivity using mantel tests is typically done using precise geographic locations, which was not possible for the natal origins of the painted ladies given the imprecision of isotope-based geographic assignment. However, the centroid of the highly probable natal origin should provide a good estimate of the relative longitude of the actual natal origin (i.e., if the painted lady originated from the east or west).

## Supplemental information

Document S1. Figures S1–S11 and Table S1

Table S2. Capture date and location of each painted lady sample and the hydrogen isotope value (ẟ2H ± sd) and strontium isotope ratio (87Sr/86Sr ± sd) measured in the wing tissue (n = 118) For additional metadata, see the Key Resources Table, related to STAR Methods

## References

[1] Chapman J.W., Reynolds D.R., Wilson K. (2015). Long-range seasonal migration in insects: Mechanisms, evolutionary drivers and ecological consequences. Ecol. Lett..

[2] Chowdhury S., Zalucki M.P., Amano T., Woodworth B.K., Venegas-Li R., Fuller R.A. (2021). Seasonal spatial dynamics of butterfly migration. Ecol. Lett..

[3] Menchetti M., Guéguen M., Talavera G. (2019). Spatio-temporal ecological niche modelling of multigenerational insect migrations. Proc. R. Soc. A B..

[4] García-Berro A., Talla V., Vila R., Wai H.K., Shipilina D., Chan K.G., Pierce N.E., Backström N., Talavera G. (2023). Migratory behaviour is positively associated with genetic diversity in butterflies. Mol. Ecol..

[5] Gao B., Hedlund J., Reynolds D.R., Zhai B., Hu G., Chapman J.W. (2020). The ‘migratory connectivity’ concept, and its applicability to insect migrants. Mov. Ecol..

[6] Gilroy J.J., Gill J.A., Butchart S.H.M., Jones V.R., Franco A.M.A. (2016). Migratory diversity predicts population declines in birds. Ecol. Lett..

[7] Zylstra E.R., Ries L., Neupane N., Saunders S.P., Ramírez M.I., Rendón-Salinas E., Oberhauser K.S., Farr M.T., Zipkin E.F. (2021). Changes in climate drive recent monarch butterfly dynamics. Nat. Ecol. Evol..

[8] Salih A.A.M., Baraibar M., Mwangi K.K., Artan G. (2020). Climate change and locust outbreak in East Africa. Nat. Clim. Chang..

[9] Lv H., Zhai M.Y., Zeng J., Zhang Y.Y., Zhu F., Shen H.M., Qiu K., Gao B.Y., Reynolds D.R., Chapman J.W., Hu G. (2023). Changing patterns of the East Asian monsoon drive shifts in migration and abundance of a globally important rice pest. Glob. Chang. Biol..

[10] Lok T., Overdijk O., Piersma T. (2015). The cost of migration: Spoonbills suffer higher mortality during trans-Saharan spring migrations only. Biol. Lett..

[11] Klaassen R.H.G., Hake M., Strandberg R., Koks B.J., Trierweiler C., Exo K.-M., Bairlein F., Alerstam T. (2014). When and where does mortality occur in migratory birds? Direct evidence from long-term satellite tracking of raptors. J. Anim. Ecol..

[12] Guilherme J.L., Jones V.R., Catry I., Beal M., Dias M.P., Oppel S., Vickery J.A., Hewson C.M., Butchart S.H.M., Rodrigues A.S.L. (2023). Connectivity between countries established by landbirds and raptors migrating along the African–Eurasian flyway. Conserv. Biol..

[13] Fattorini N., Costanzo A., Romano A., Rubolini D., Baillie S., Bairlein F., Spina F., Ambrosini R. (2023). Eco-evolutionary drivers of avian migratory connectivity. Ecol. Lett..

[14] Sanderson F.J., Donald P.F., Pain D.J., Burfield I.J., van Bommel F.P. (2006). Long-term population declines in Afro-Palearctic migrant birds. Biol. Conserv..

[15] Marcacci G., Briedis M., Diop N., Diallo A.Y., Kebede F., Jacot A. (2023). A roadmap integrating research, policy, and actions to conserve Afro-Palearctic migratory landbirds at a flyway scale. Conserv. Lett..

[16] Vickery J.A., Ewing S.R., Smith K.W., Pain D.J., Bairlein F., Škorpilová J., Gregory R.D. (2014). The decline of Afro-Palaearctic migrants and an assessment of potential causes. Ibis.

[17] Satterfield D.A., Sillett T.S., Chapman J.W., Altizer S., Marra P.P. (2020). Seasonal insect migrations: Massive, influential, and overlooked. Front. Ecol. Environ..

[18] Reich M.S., Shipilina D., Talla V., Bahleman F., Kébé K., Berger J.L., Backström N., Talavera G., Bataille C.P. (2023). Isotope geolocation and population genomics in *Vanessa cardui:* Short- and long-distance migrants are genetically undifferentiated. bioRxiv.

[19] Talavera G., García-Berro A., Talla V.N.K., Ng’iru I., Bahleman F., Kébé K., Nzala K.M., Plasencia D., Marafi M.A.J., Kassie A. (2023). The Afrotropical breeding grounds of the Palearctic-African migratory painted lady butterflies (*Vanessa cardui*). Proc. Natl. Acad. Sci. USA.

[20] Suchan T., Talavera G., Sáez L., Ronikier M., Vila R. (2018). Pollen metabarcoding as a tool for tracking long-distance insect migrations. Mol. Ecol. Resour..

[21] Talavera G., Bataille C., Benyamini D., Gascoigne-Pees M., Vila R. (2018). Round-trip across the Sahara: Afrotropical painted lady butterflies recolonize the Mediterranean in early spring. Biol. Lett..

[22] Stefanescu C., Soto D.X., Talavera G., Vila R., Hobson K.A. (2016). Long-distance autumn migration across the Sahara by painted lady butterflies: exploiting resource pulses in the tropical savannah. Biol. Lett..

[23] Talavera G., Vila R. (2016). Discovery of mass migration and breeding of the painted lady butterfly *Vanessa cardui* in the Sub-Sahara: The Europe-Africa migration revisited. Biol. J. Linn. Soc. Lond..

[24] Shipilina D., Höök L., Näsvall K., Talla V., Palahí A., Parkes E., Vila R., Talavera G., Backström N. (2024). Gene expression responses to environmental cues shed light on components of the migratory syndrome in butterflies. bioRxiv.

[25] Nasvall K., Shipilina D., Vila R., Talavera G., Backstrom N. (2023). Resource availability affects activity profiles of regulatory elements in a long-distance butterfly migrant. TechRxiv.

[26] Stefanescu C., Ubach A., Wiklund C. (2021). Timing of mating, reproductive status and resource availability in relation to migration in the painted lady butterfly. Anim. Behav..

[27] Stefanescu C., Páramo F., Åkesson S., Alarcón M., Ávila A., Brereton T., Carnicer J., Cassar L.F., Fox R., Heliölä J. (2013). Multi-generational long-distance migration of insects: studying the painted lady butterfly in the Western Palaearctic. Ecography.

[28] Hammad, S.M., El-Minshawy, A.M., and Raafat, A.M. (1973). Biology of the Painted Lady Vanessa (Pyrameis Cardui L. Lepidoptera, Nymphalidae) on Artichoke in A.R. Of Egypt. 2nd Italian Congr Int. Carc. Bari. Italy, 929–935.

[29] Diffendorfer J.E., Drum R.G., Mitchell G.W., Rendón-Salinas E., Sánchez-Cordero V., Semmens D.J., Thogmartin W.E., March I.J. (2023). The benefits of big-team science for conservation: Lessons learned from trinational monarch butterfly collaborations. Front. Environ. Sci..

[30] Flockhart D.T.T., Kyser T.K., Chipley D., Miller N.G., Norris D.R. (2015). Experimental evidence shows no fractionation of strontium isotopes (^87^Sr/^86^Sr) among soil, plants, and herbivores: implications for tracking wildlife and forensic science. Isotopes Environ. Health Stud..

[31] Hobson K.A., Wassenaar L.I., Taylor O.R. (1999). Stable isotopes (δD and δ^13^C) are geographic indicators of natal origins of monarch butterflies in eastern North America. Oecologia.

[32] Lindroos E.E., Bataille C.P., Holder P.W., Talavera G., Reich M.S. (2023). Temporal stability of δ^2^H in insect tissues: Implications for isotope-based geographic assignments. Front. Ecol. Evol..

[33] Reich M.S., Kindra M., Dargent F., Hu L., Flockhart D.T.T., Norris D.R., Kharouba H., Talavera G., Bataille C.P. (2023). Metals and metal isotopes incorporation in insect wings: Implications for geolocation and pollution exposure. Front. Ecol. Evol..

[34] Wassenaar L.I., Hobson A. (1998). Natal origins of migratory monarch butterflies at wintering colonies in Mexico: New isotopic evidence. Proc. Natl. Acad. Sci. USA.

[35] Bowen G.J., Wassenaar L.I., Hobson K.A. (2005). Global application of stable hydrogen and oxygen isotopes to wildlife forensics. Oecologia.

[36] Reich M.S., Flockhart D.T.T., Norris D.R., Hu L., Bataille C.P. (2021). Continuous-surface geographic assignment of migratory animals using strontium isotopes: A case study with monarch butterflies. Methods Ecol. Evol..

[37] Ghouri S., Reich M.S., Lopez-Mañas R., Talavera G., Bowen G.J., Vila R., Talla V.N.K., Collins S.C., Martins D.J., Bataille C.P. (2024). A hydrogen isoscape for tracing the migration of herbivorous lepidopterans across the Afro-Palearctic range. Rapid Comm Mass Spectrometry.

[38] Bataille C.P., Crowley B.E., Wooller M.J., Bowen G.J. (2020). Advances in global bioavailable strontium isoscapes. Palaeogeogr. Palaeoclimatol. Palaeoecol..

[39] Bataille C.P., Von Holstein I.C.C., Laffoon J.E., Willmes M., Liu X.-M., Davies G.R. (2018). A bioavailable strontium isoscape for Western Europe: A machine learning approach. PLoS One.

[40] Le Corre M., Dargent F., Grimes V., Wright J., Côté S.D., Reich M.S., Candau J.-N., Miller M., Holmes B., Bataille C.P., et al. An ensemble machine learning bioavailable strontium isoscape for Eastern Canada. FACETS. 10.1139/facets-2024-0180.

[41] Copeland S.R., Sponheimer M., de Ruiter D.J., Lee-Thorp J.A., Codron D., le Roux P.J., Grimes V., Richards M.P. (2011). Strontium isotope evidence for landscape use by early hominins. Nature.

[42] Wang X., Bocksberger G., Lautenschläger T., Finckh M., Meller P., O’Malley G.E., Oelze V.M. (2023). A bioavailable strontium isoscape of Angola with implications for the archaeology of the transatlantic slave trade. J. Archaeol. Sci..

[43] Degryse P., De Muynck D., Delporte S., Boyen S., Jadoul L., De Winne J., Ivaneanu T., Vanhaecke F. (2012). Strontium isotopic analysis as an experimental auxiliary technique in forensic identification of human remains. Anal. Methods.

[44] Pye K. (2004). Isotope and trace element analysis of human teeth and bones for forensic purposes. SP (Sci. Prog.).

[45] Van Der Merwe N.J., Lee-Thorp J.A., Thackeray J.F., Hall-Martin A., Kruger F.J., Coetzee H., Bell R.H.V., Lindeque M. (1990). Source-area determination of elephant ivory by isotopic analysis. Nature.

[46] Dinerstein E., Olson D., Joshi A., Vynne C., Burgess N.D., Wikramanayake E., Hahn N., Palminteri S., Hedao P., Noss R. (2017). An ecoregion-based approach to protecting half the terrestrial realm. Bioscience.

[47] Larsen T.B. (1975). Provisional notes of migrant butterflies in Lebanon. Atalanta.

[48] Marra P.P., Studds C.E., Webster M., Breed M.D., Moore J. (2010). Encyclopedia of Animal Behavior.

[49] Webster M.S., Marra P.P., Haig S.M., Bensch S., Holmes R.T. (2002). Links between worlds: unraveling migratory connectivity. Trends Ecol. Evol..

[50] Koleček J., Procházka P., El-Arabany N., Tarka M., Ilieva M., Hahn S., Honza M., De La Puente J., Bermejo A., Gürsoy A. (2016). Cross-continental migratory connectivity and spatiotemporal migratory patterns in the great reed warbler. J. Avian Biol..

[51] Finch T., Saunders P., Avilés J.M., Bermejo A., Catry I., De La Puente J., Emmenegger T., Mardega I., Mayet P., Parejo D. (2015). A pan-European, multipopulation assessment of migratory connectivity in a near-threatened migrant bird. Diversity Distrib.

[52] Briedis M., Bauer S., Adamík P., Alves J.A., Costa J.S., Emmenegger T., Gustafsson L., Koleček J., Krist M., Liechti F. (2020). Broad-scale patterns of the Afro-Palaearctic landbird migration. Glob. Ecol. Biogeogr..

[53] Jiguet F., Burgess M., Thorup K., Conway G., Arroyo Matos J.L., Barber L., Black J., Burton N., Castelló J., Clewley G. (2019). Desert crossing strategies of migrant songbirds vary between and within species. Sci. Rep..

[54] Åkesson S., Bianco G., Hedenström A. (2016). Negotiating an ecological barrier: Crossing the Sahara in relation to winds by common swifts. Philos. Trans. R. Soc. B.

[55] Marx M., Korner-Nievergelt F., Quillfeldt P. (2016). Analysis of ring recoveries of European Turtle Doves *Streptopelia turtur* — Flyways, migration timing and origin areas of hunted birds. Acta Ornithol. (Warszaw).

[56] Suchan T., Bataille C.P., Reich M.S., Toro-Delgado E., Vila R., Pierce N.E., Talavera G. (2024). A trans-oceanic flight of over 4,200 km by painted lady butterflies. Nat. Commun..

[57] Tessnow A.E., Nagoshi R.N., Meagher R.L., Fleischer S.J. (2023). Revisiting fall armyworm population movement in the United States and Canada. Front. Insect Sci..

[58] Gorki J.L., López-Mañas R., Sáez L., Menchetti M., Shapoval N., Andersen A., Benyamini D., Daniels S., García-Berro A., Reich M.S. (2024). Pollen metabarcoding reveals the origin and multigenerational migratory pathway of an intercontinental-scale butterfly outbreak. Curr. Biol..

[59] Hawkes W.L.S., Walliker E., Gao B., Forster O., Lacey K., Doyle T., Massy R., Roberts N.W., Reynolds D.R., Özden Ö. (2022). Huge spring migrations of insects from the Middle East to Europe: Quantifying the migratory assemblage and ecosystem services. Ecography.

[60] López-Mañas R., Pascual-Díaz J.P., García-Berro A., Bahleman F., Reich M.S., Pokorny L., Bataille C.P., Vila R., Domingo-Marimon C., Talavera G. (2022). Erratic spatiotemporal vegetation growth anomalies drive population outbreaks in a trans-Saharan insect migrant. Proc. Natl. Acad. Sci..

[61] Somveille M., Manica A., Rodrigues A.S.L. (2019). Where the wild birds go: Explaining the differences in migratory destinations across terrestrial bird species. Ecography.

[62] Pelton E.M., Schultz C.B., Jepsen S.J., Black S.H., Crone E.E. (2019). Western monarch population plummets: Status, probable causes, and recommended conservation actions. Front. Ecol. Evol..

[63] Vandenbosch R. (2007). What do monarch population time series tell us about eastern and western population mixing?. J. Lepid. Soc..

[64] Stefanescu C., Alarcón M., Izquierdo R., Páramo F., Àvila A. (2011). Moroccan source areas of the painted lady butterfly *Vanessa cardui* (Nymphalidae: Nymphalinae) migrating into Europe in spring. J. Lepid. Soc..

[65] Flockhart D.T.T., Wassenaar L.I., Martin T.G., Hobson K.A., Wunder M.B., Norris D.R. (2013). Tracking multi-generational colonization of the breeding grounds by monarch butterflies in eastern North America. Proc. Biol. Sci..

[66] Goehring L., Oberhauser K.S. (2002). Effects of photoperiod, temperature, and host plant age on induction of reproductive diapause and development time in Danaus plexippus. Ecol. Entomol..

[67] Aikens E.O., Mysterud A., Merkle J.A., Cagnacci F., Rivrud I.M., Hebblewhite M., Hurley M.A., Peters W., Bergen S., De Groeve J. (2020). Wave-like patterns of plant phenology determine ungulate movement tactics. Curr. Biol..

[68] Hurme E., Fahr J., Eric-Moise B.F., Eric-Moise B.F., O'Mara M.T., O’Mara M.T., Tanshi I., Webala P.W., Weber N., Wikelski M., Dechmann D.K.N. (2022). Fruit bat migration matches green wave in seasonal landscapes. Funct. Ecol..

[69] Wang X., Cao L., Fox A.D., Fuller R., Griffin L., Mitchell C., Zhao Y., Moon O.-K., Cabot D., Xu Z. (2019). Stochastic simulations reveal few green wave surfing populations among spring migrating herbivorous waterfowl. Nat. Commun..

[70] Franzoi A., Larsen S., Franceschi P., Hobson K.A., Pedrini P., Camin F., Bontempo L. (2021). Multidimensional natal isotopic niches reflect migratory patterns in birds. Sci. Rep..

[71] Cardenas-Ortiz L., Bayly N.J., Kardynal K.J., Hobson K.A. (2020). Defining catchment origins of a geographical bottleneck: Implications of population mixing and phenological overlap for the conservation of Neotropical migratory birds. Condor.

[72] Nelson D.M., Braham M., Miller T.A., Duerr A.E., Cooper J., Lanzone M., Lemaître J., Katzner T. (2015). Stable hydrogen isotopes identify leapfrog migration, degree of connectivity, and summer distribution of Golden Eagles in eastern North America. Condor.

[73] Hobson K.A., Kusack J.W., Mora-Alvarez B.X. (2021). Origins of six species of butterflies migrating through Northeastern Mexico: New insights from stable isotope (δ^2^H) analyses and a call for documenting butterfly migrations. Diversity.

[74] Holmgren N., Lundberg S. (1993). Despotic behaviour and the evolution of migration patterns in birds. Ornis Scand..

[75] Ethier D.M., Mitchell G.W. (2023). Effects of climate on fall migration phenology of monarch butterflies departing the northeastern breeding grounds in Canada. Glob. Chang. Biol..

[76] Guerra P.A., Reppert S.M. (2013). Coldness triggers northward flight in remigrant monarch butterflies. Curr. Biol..

[77] Newton I. (2007). Weather-related mass-mortality events in migrants. Ibis.

[78] Morrison C.A., Alves J.A., Gunnarsson T.G., þórisson B., Gill J.A. (2019). Why do earlier-arriving migratory birds have better breeding success?. Ecol. Evol..

[79] Larsen T.B. (1984). The zoogeographical composition and distribution of the Arabian butterflies (Lepidoptera; Rhopalocera). J. Biogeogr..

[80] Larsen T.B. (1982). The importance of migration to the butterfly fauna of Arabia (Lep., Rhopalocera). Atalanta.

[81] Borisov S.N., Iakovlev I.K., Borisov A.S., Ganin M.Y., Tiunov A.V. (2020). Seasonal migrations of *Pantala flavescens* (Odonata : Libellulidae) in Middle Asia and understanding of the migration model in the Afro-Asian region using stable isotopes of hydrogen. Insects.

[82] Hobson K.A., Anderson R.C., Soto D.X., Wassenaar L.I. (2012). Isotopic evidence that dragonflies (*Pantala flavescens*) migrating through the Maldives come from the Northern Indian subcontinent. PLoS One.

[83] Biebach H., Friedrich W., Heine G. (1986). Interaction of bodymass, fat, foraging and stopover period in trans-Sahara migrating passerine birds. Oecologia.

[84] Hallworth M.T., Marra P.P., McFarland K.P., Zahendra S., Studds C.E. (2018). Tracking dragons: Stable isotopes reveal the annual cycle of a long-distance migratory insect. Biol. Lett..

[85] R Core Team (2021).

[86] Magozzi S., Bataille C.P., Hobson K.A., Wunder M.B., Howa J.D., Contina A., Vander Zanden H.B., Bowen G.J. (2021). Calibration chain transformation improves the comparability of organic hydrogen and oxygen stable isotope data. Methods Ecol. Evol..

[87] Ma C., Vander Zanden H.B., Wunder M.B., Bowen G.J. (2020). assignR: An R package for isotope-based geographic assignment. Methods Ecol. Evol..

[88] Hobson K.A., Plint T., Serrano E.G., Alvarez X.M., Ramirez I., Longstaffe F.J. (2017). Within-wing isotopic (δ^2^H, δ^13^C, δ^15^N) variation of monarch butterflies: implications for studies of migratory origins and diet. Anim. Migrat..

[89] Paritte J.M., Kelly J.F. (2009). Effect of cleaning regime on stable-isotope ratios of feathers in Japanese quail (*Coturnix japonica*). Auk.

[90] Meier-Augenstein W., Chartrand M.M.G., Kemp H.F., St-Jean G. (2011). An inter-laboratory comparative study into sample preparation for both reproducible and repeatable forensic ^2^H isotope analysis of human hair by continuous flow isotope ratio mass spectrometry. Rapid Commun. Mass Spectrom..

[91] Gehre M., Renpenning J., Gilevska T., Qi H., Coplen T.B., Meijer H.A.J., Brand W.A., Schimmelmann A. (2015). On-Line hydrogen-isotope measurements of organic samples using elemental chromium: An extension for high temperature elemental-analyzer techniques. Anal. Chem..

[92] Wassenaar L.I., Hobson K.A. (2003). Comparative equilibration and online technique for determination of non-exchangeable hydrogen of keratins for use in animal migration studies. Isotopes Environ. Health Stud..

[93] Bataille C.P., Ammer S.T.M., Bhuiyan S., Chartrand M.M.G., St-Jean G., Bowen G.J. (2022). Multi-isotopes in human hair: A tool to initiate cross-border collaboration in international cold-cases. PLoS One.

[94] Soto D.X., Koehler G., Wassenaar L.I., Hobson K.A. (2017). Re-evaluation of the hydrogen stable isotopic composition of keratin calibration standards for wildlife and forensic science applications. Rapid Commun. Mass Spectrom..

[95] Coplen T.B., Qi H. (2012). USGS42 and USGS43: Human-hair stable hydrogen and oxygen isotopic reference materials and analytical methods for forensic science and implications for published measurement results. Forensic Sci. Int..

[96] Hu L., Chartrand M.M.G., St-Jean G., Lopes M., Bataille C.P. (2020). Assessing the reliability of mobility interpretation from a multi-isotope hair profile on a traveling individual. Front. Ecol. Evol..

[97] Moore L.J., Murphy T.J., Barnes I.L., Paulsen P.J. (1982). Absolute isotopic abundance ratios and atomic weight of a reference sample of strontium. J. Res. Natl. Bur. Stand..

[98] Das S., Miller B.V., Prospero J., Chellam S. (2022). Sr-Nd-Hf isotopic analysis of reference materials and natural and anthropogenic particulate matter sources: Implications for accurately tracing North African dust in complex urban atmospheres. Talanta.

[99] Capo R.C., Stewart B.W., Chadwick O.A. (1998). Strontium isotopes as tracers of ecosystem processes: Theory and methods. Geoderma.

[100] Serna A., Prates L., Mange E., Salazar-García D.C., Bataille C.P. (2020). Implications for paleomobility studies of the effects of quaternary volcanism on bioavailable strontium: A test case in North Patagonia (Argentina). J. Archaeol. Sci..

[101] Chien C., Mackey K.R.M., Dutkiewicz S., Mahowald N.M., Prospero J.M., Paytan A. (2016). Effects of African dust deposition on phytoplankton in the western tropical Atlantic Ocean off Barbados. Global Biogeochem. Cycles.

[102] Genuer R., Poggi J.-M., Tuleau-Malot C. (2015). VSURF: An R Package for Variable Selection Using Random Forests. R J..

[103] Kramer R.T., Kinaston R.L., Holder P.W., Armstrong K.F., King C.L., Sipple W.D.K., Martin A.P., Pradel G., Turnbull R.E., Rogers K.M. (2022). A bioavailable strontium (^87^Sr/^86^Sr) isoscape for Aotearoa New Zealand: Implications for food forensics and biosecurity. PLoS One.

[104] Janzen A., Bataille C., Copeland S.R., Quinn R.L., Ambrose S.H., Reed D., Hamilton M., Grimes V., Richards M.P., le Roux P., Roberts P. (2020). Spatial variation in bioavailable strontium isotope ratios (^87^Sr/^86^Sr) in Kenya and northern Tanzania: Implications for ecology, paleoanthropology, and archaeology. Palaeogeogr. Palaeoclimatol. Palaeoecol..

[105] Bataille C.P., Bowen G.J. (2012). Mapping ^87^Sr/^86^Sr variations in bedrock and water for large scale provenance studies. Chem. Geol..

[106] Hartman G., Richards M. (2014). Mapping and defining sources of variability in bioavailable strontium isotope ratios in the Eastern Mediterranean. Geochem. Cosmochim. Acta.

[107] Kuhn M. (2008). Building predictive models in *R* using the caret package. J. Stat. Soft..

[108] Hengl T., Nussbaum M., Wright M.N., Heuvelink G.B.M., Gräler B. (2018). Random forest as a generic framework for predictive modeling of spatial and spatio-temporal variables. PeerJ.

[109] Georganos S., Grippa T., Niang Gadiaga A., Linard C., Lennert M., Vanhuysse S., Mboga N., Wolff E., Kalogirou S. (2021). Geographical random forests: a spatial extension of the random forest algorithm to address spatial heterogeneity in remote sensing and population modelling. Geocarto Int..

[110] Hengl T., Miller M.A.E., Križan J., Shepherd K.D., Sila A., Kilibarda M., Antonijević O., Glušica L., Dobermann A., Haefele S.M. (2021). African soil properties and nutrients mapped at 30 m spatial resolution using two-scale ensemble machine learning. Sci. Rep..

[111] Lu B., Hardin J. (2021). A unified framework for random forest prediction error estimation. J. Mach. Learn. Res..

[112] Katzner T.E., Nelson D.M., Braham M.A., Doyle J.M., Fernandez N.B., Duerr A.E., Bloom P.H., Fitzpatrick M.C., Miller T.A., Culver R.C.E. (2017). Golden Eagle fatalities and the continental-scale consequences of local wind-energy generation. Conserv. Biol..

[113] Ambrosini R., Møller A.P., Saino N. (2009). A quantitative measure of migratory connectivity. J. Theor. Biol..

