# Supplementary material for: Trans-Saharan migratory patterns in Vanessa cardui and evidence for a southward leapfrog migration
Source: iScience. 2024 Nov 8;27(12):111342. doi: 10.1016/j.isci.2024.111342 (PMC11626715; doi:10.1016/j.isci.2024.111342)
Supplement: Document S1. Figures S1–S11 and Table S1 [file mmc1.pdf]

iScience, Volume *27*

Supplemental information

Trans-Saharan migratory patterns in *Vanessa cardui* and evidence for a southward leapfrog migration

Megan S. Reich, Sana Ghouri, Samantha Zabudsky, Lihai Hu, Mael Le Corre, Ivy Ng'iru, Dubi Benyamini, Daria Shipilina, Steve C. Collins, Dino J. Martins, Roger Vila, Gerard Talavera, and Clément P. Bataille

Supplemental information

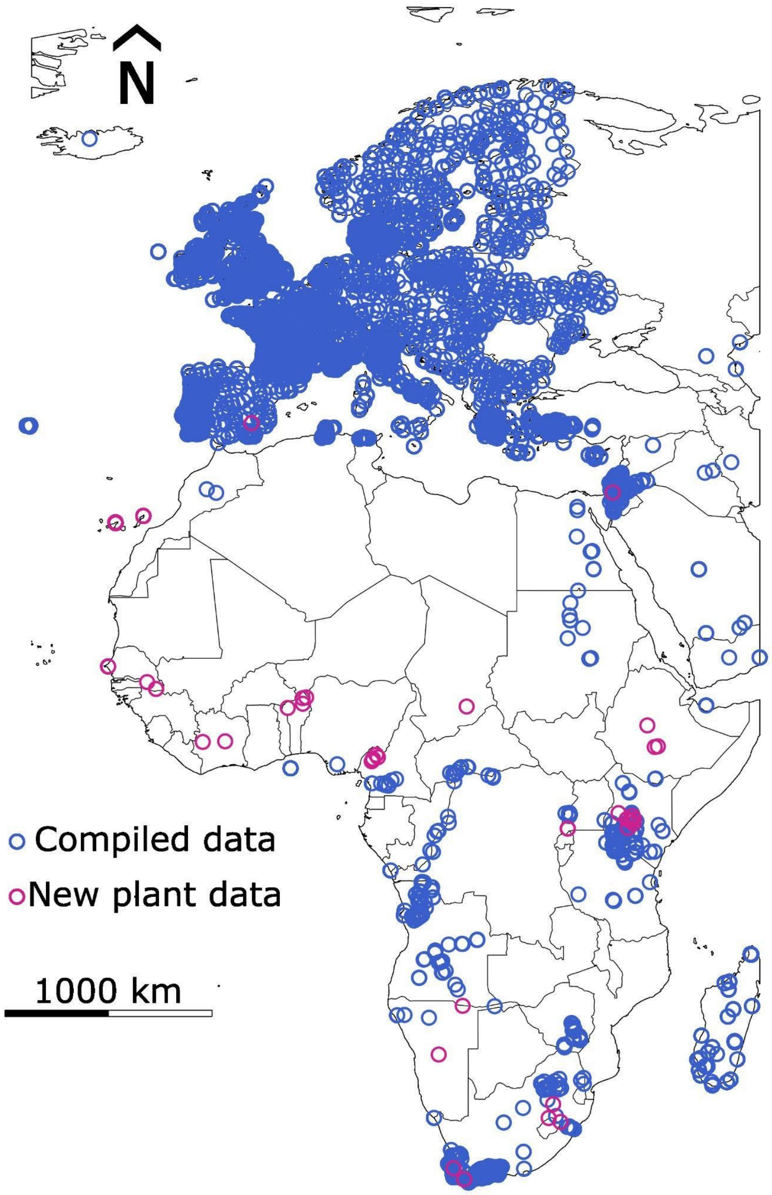

Figure S1 Geographic distribution of bioavailable $^{87}Sr/^{86}Sr$ data compilation across the Afro-Palearctic range. Compiled data from existing literature is depicted in blue, while pink represents newly collected plant sample data from this study (see Key Resources Table). The shapefile for country boundaries was obtained from the *rnaturalearth* package [S1]. Related to STAR Methods.

Table S1 List of 28 geological, climatic, environmental, and anthropogenic geospatial data used as auxiliary variables in the random forest regression and in the spatial interpolation ensemble machine learning to create our regional bioavailable strontium isoscape for the Afro-Palearctic range based on Bataille et al. [S2]. The important variables selected by *VSURF* are in bold. Related to STAR Methods.

Variable ID	Description	Source of data
**r.age**	**Terrane age attribute (Myrs)**	**[S3]**
**r.ai**	**Global Aridity Index**	**[S4]**
r.bulk	Bulk density of the fine earth fraction (kg/m^3)	[S5]
r.cec	Cation Exchange Capacity (cmol+ /kg)	[S5]
**r.clay**	**Clay (weight %)**	**[S5]**
**r.biof**	**Biofuels (Black Carbon) ($kg/m^2/s$)**	**[S6]**
r.biog	Primary biogenic deposition ($kg/m^2/s$)	[S6]
r.wet	Wet dust deposition ($kg/m^2/s$)	[S6]
**r.foss**	**Fossil Fuel (Black Carbon) ($kg/m^2/s$)**	**[S6]**
r.dry	Dry dust deposition ($kg/m^2/s$)	[S6]
**r.dust**	**Multi-models average of dust deposition ($g/m^2/year$)**	**[S6]**
r.dust20	Dust deposition ($kg/m^2/s$)	[S6]
**r.fire**	**Black carbon deposition ($kg/m^2/s$)**	**[S6]**
**r.volc**	**Volcanoes (S) ($kg/m^2/s$)**	**[S7]**
r.elevation	Hole-filled Digital Elevation Model	[S8]
r.GUM	Unconsolidated sediment map	[S9]
r.bouger	WGM2012_Bouguer Mean	[S10]
**r.m1**	**Median bedrock model ($^{87}Sr/^{86}Sr$)**	**[S11]**
**r.mat**	**Mean annual temperature (°C)**	**[S12]**
r.map	Mean annual precipitation (mm/yr)	[S12]
r.maxage_geol	GLiM age attribute – maximum (Myrs)	[S2,S13]
**r.meanage_geol**	**GLiM age attribute – mean (Myrs)**	**[S2,S13]**
r.minage_geol	GLiM age attribute – minimum (Myrs)	[S2,S13]
**r.pet**	**Global Potential Evapo-Transpiration (mm day^{-1})**	**[S4]**
**r.ph**	**Soil pH in H_2O solution (x10)**	**[S5]**
**r.salt**	**Simulation of sea salt aerosol deposition ($g/m^2/year$)**	**[S6]**
r.srsrq1	Quartile 1 bedrock model ($^{87}Sr/^{86}Sr$)	[S11]
r.srsrq3	Quartile 3 bedrock model ($^{87}Sr/^{86}Sr$)	[S11]

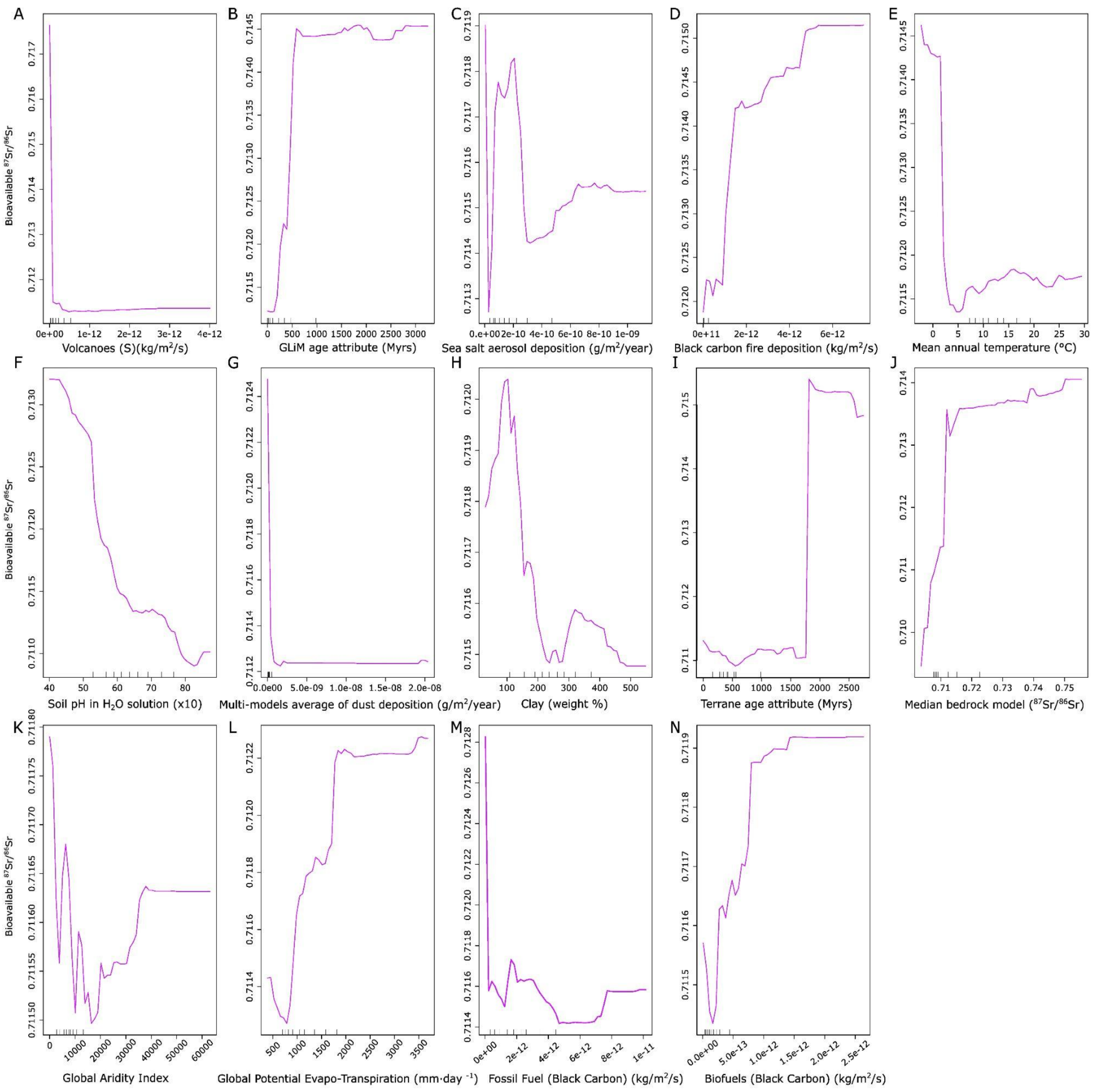

Figure S2 Partial dependence plots between each predictor (x-axis) and the predicted bioavailable $^{87}Sr/^{86}Sr$ (y-axis) from the random forest regression. Hash marks along the x-axis show sample decile values. Refer to Table S1 for the source of each variable: **(A)** volcanoes, **(B)** GLiM age attribute - mean, **(C)** sea salt aerosol deposition, **(D)** black carbon - fire deposition, **(E)** mean annual temperature, **(F)** soil pH in H_2O solution (x10), **(G)** multi-model average of dust deposition, **(H)** clay, **(I)** terrane age attribute, **(J)** median bedrock model, **(K)** global aridity index, **(L)** global potential evapotranspiration, **(M)** fossil fuel (black carbon), and **(N)** biofuels (black carbon). Related to STAR Methods.

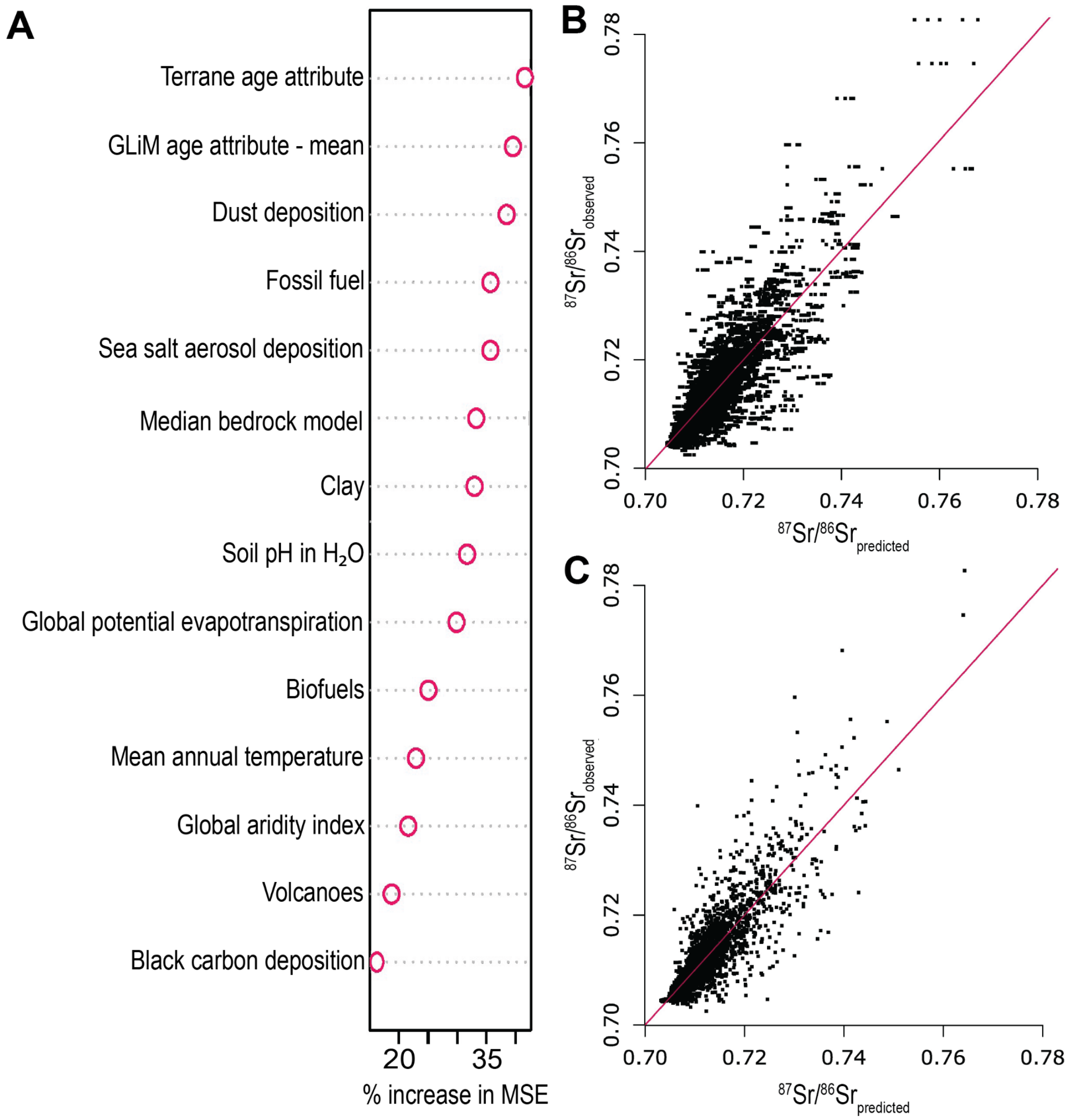

Figure S3 Machine learning model performance. **(A)** Variable importance plot after selection of predictors by *VSURF* with % increase in MSE (percent increase in mean squared error) as the evaluation standard. If a variable is important, then the model's mean squared error of prediction will increase when the variable is taken out of the model. **(B)** N-fold cross-validation results between observed and predicted bioavailable $^{87}Sr/^{86}Sr$ with the best fit linear model (pink line; RMSE = 0.0031, $R^2 = 0.73$) for the random forest regression model using the framework of Bataille et al. [S2]; **(C)** N-fold cross-validation results for the spatial interpolation ensemble machine learning model using the *landmap* package [S5]. The pink line represents the best-fit linear model (RMSE = 0.0029, $R^2 = 0.74$). Related to STAR Methods.

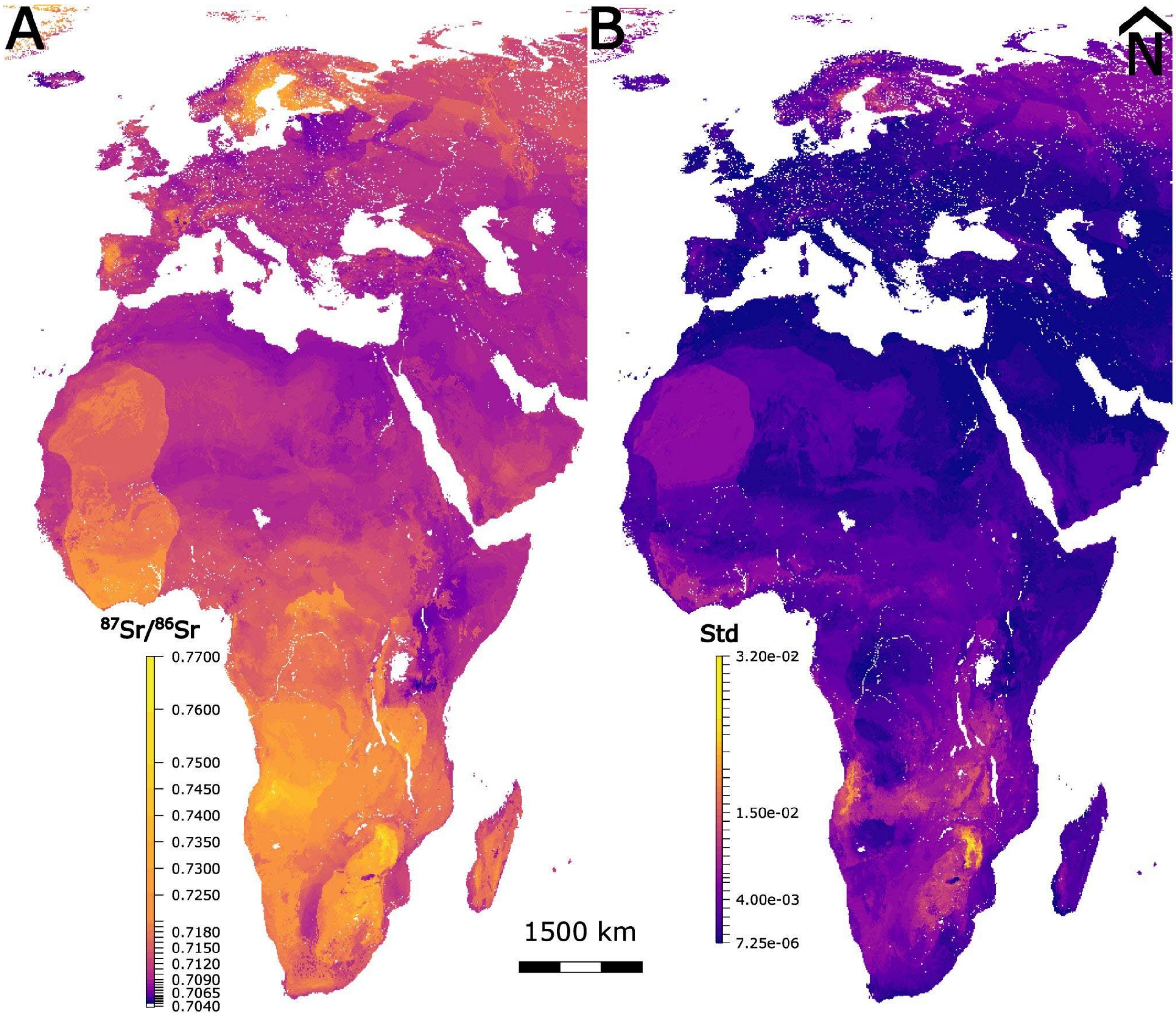

Figure S4 Predicted bioavailable strontium isoscape across the Afro-Palearctic and its associated uncertainty generated using random forest regression. **(A)** Mean bioavailable $^{87}Sr/^{86}Sr$ predictions; **(B)** Standard deviation of bioavailable $^{87}Sr/^{86}Sr$ predictions. Colour scale breakpoints were selected to improve visualisation and increase contrast. Related to STAR Methods.

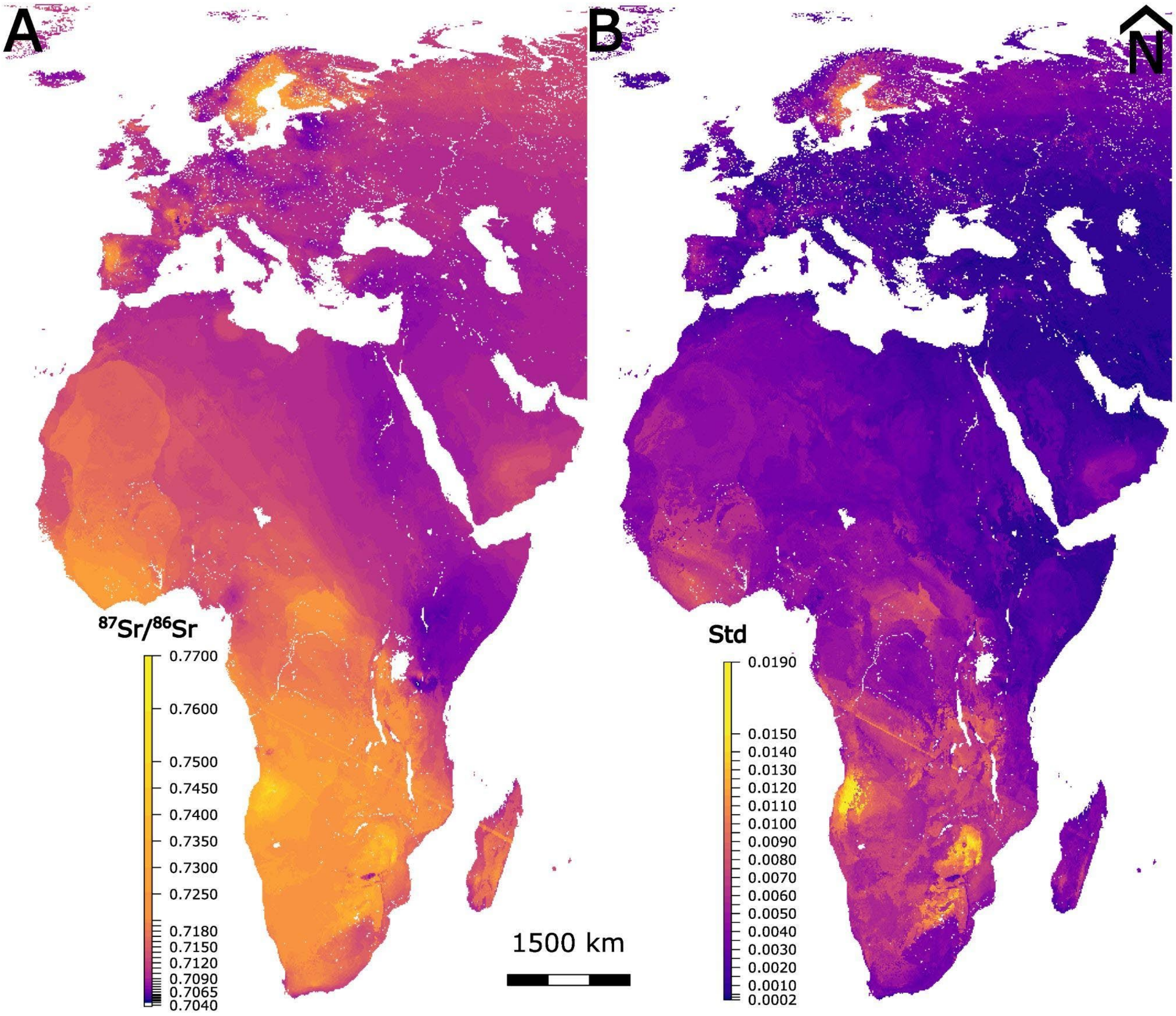

Figure S5 Predicted bioavailable strontium isoscape across the Afro-Palearctic and its associated uncertainty generated using ensemble machine learning. **(A)** Mean bioavailable $^{87}Sr/^{86}Sr$ predictions; **(B)** Standard-deviation of bioavailable $^{87}Sr/^{86}Sr$ predictions. Related to STAR Methods.

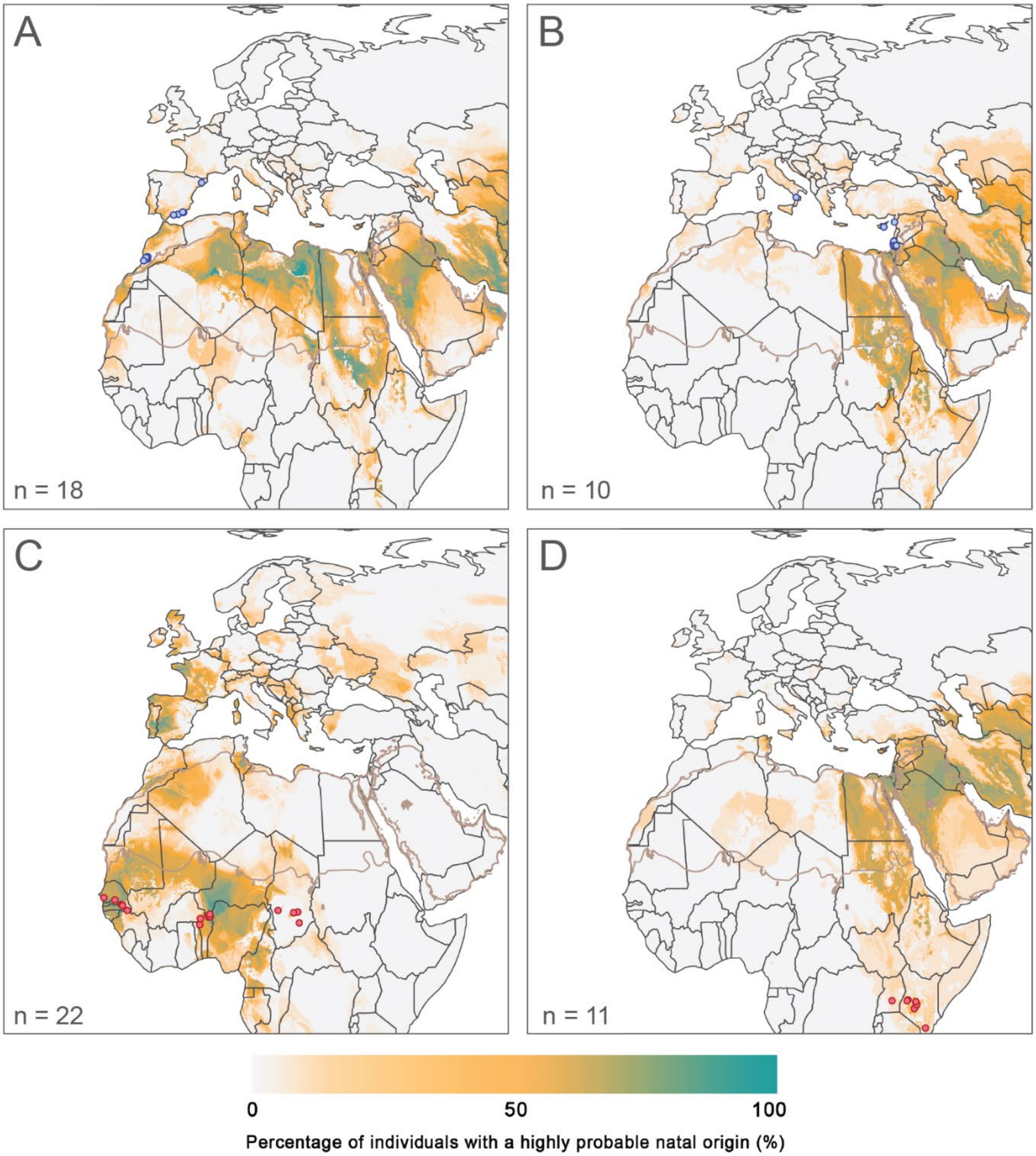

Figure S6 Stacked maps of painted ladies categorised as putative locals. **(A)** Captures from northwest of the Sahara in the late winter/spring (n =18); **(B)** captures from northeast of the Sahara in the spring (n = 10); **(C)** captures from southwest of the Sahara in the autumn (n =22); **(D)** captures from southeast of the Sahara in the late autumn (n =11). Related to Figure 2.

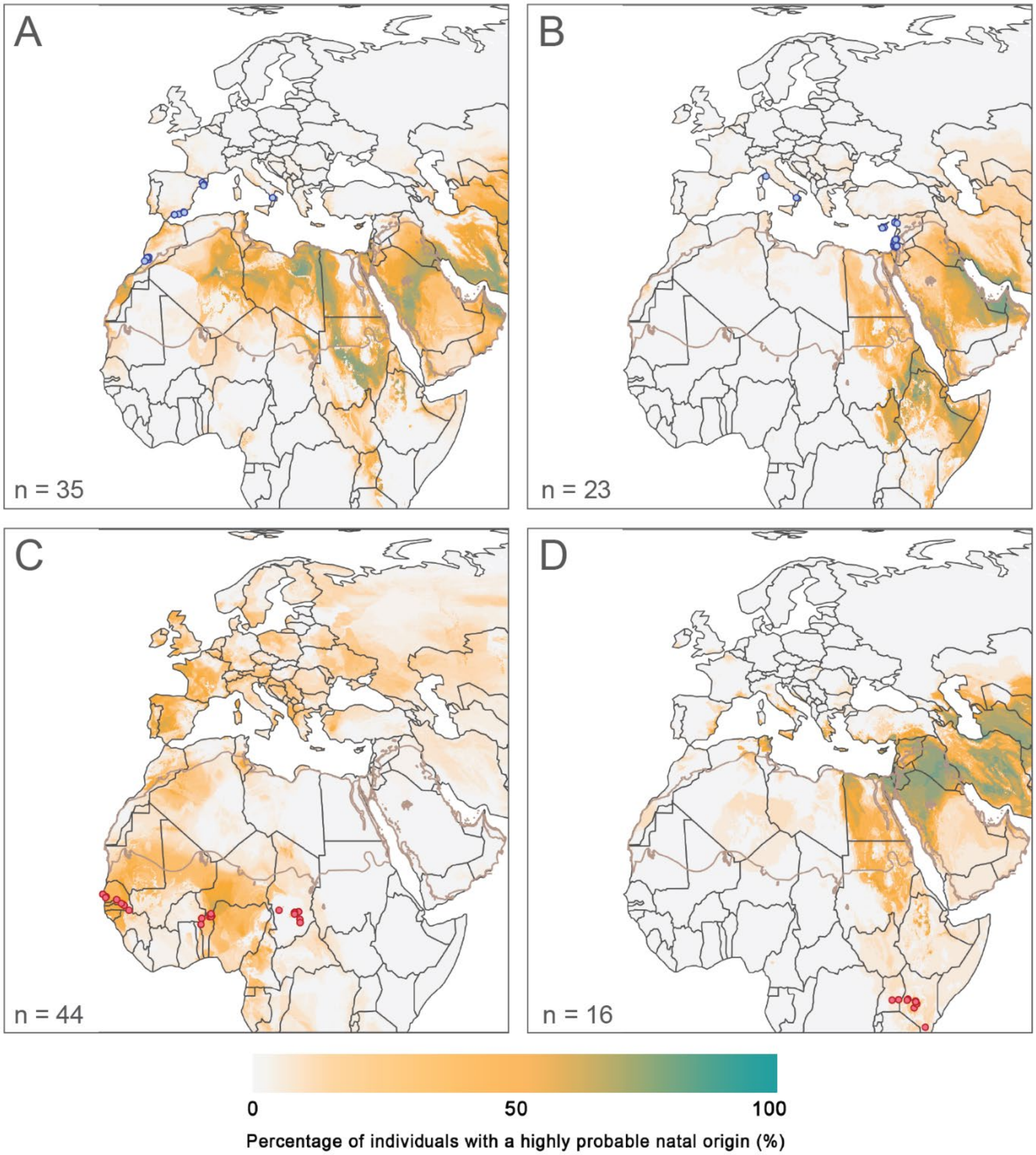

Figure S7 Stacked maps of all individuals (i.e., putative locals and migrants). **(A)** Captures from northwest of the Sahara in the late winter/spring (n = 35); **(B)** captures from northeast of the Sahara in the spring (n = 23); **(C)** captures from southwest of the Sahara in the autumn (n = 44); **(D)** captures from southeast of the Sahara in the late autumn (n = 16). Related to Figure 2.

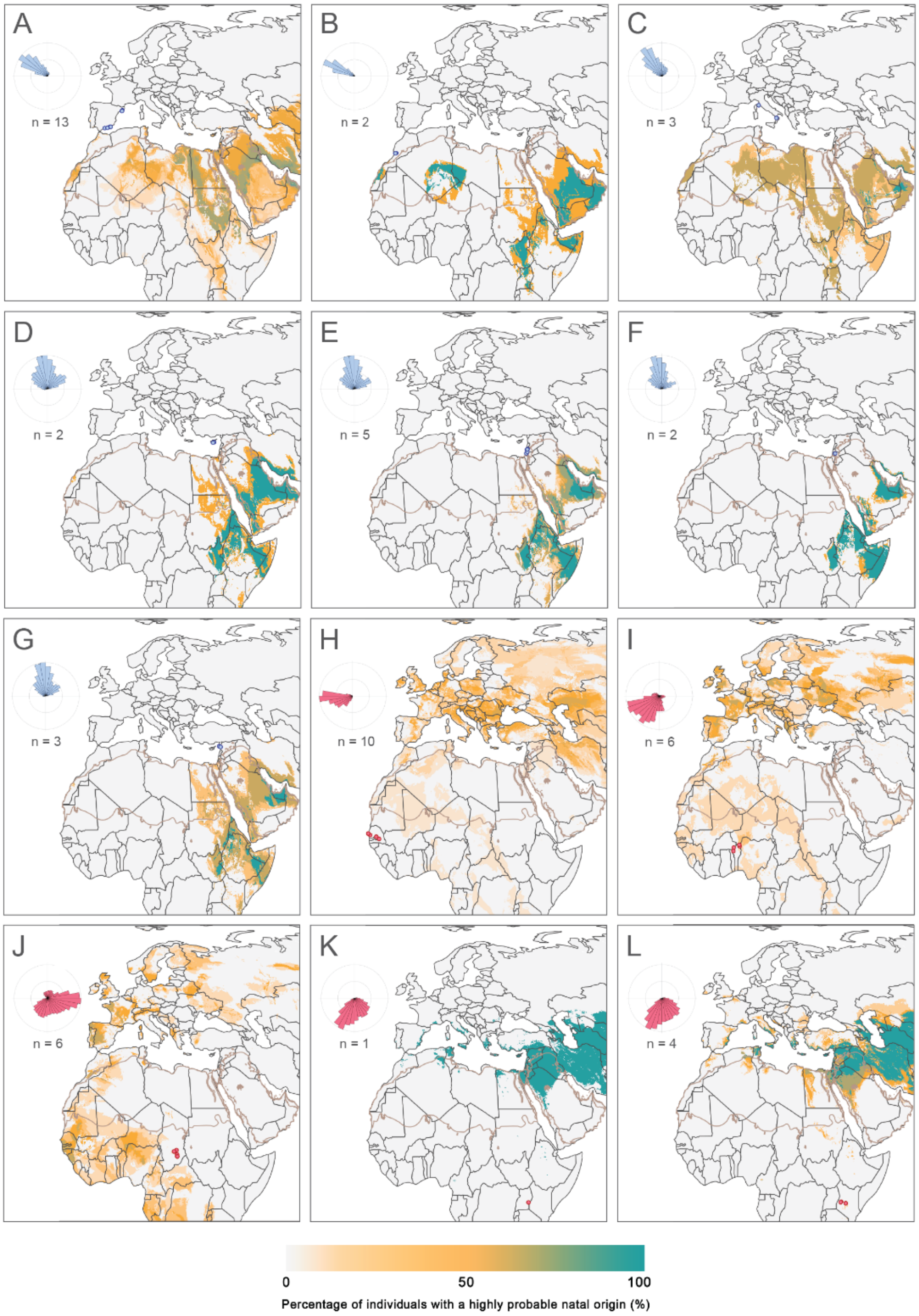

Figure S8 Stacked maps of painted ladies categorized as migratory from the δ^2H and $^{87}Sr/^{86}Sr$-based geographic assignment grouped by country of capture, illustrating the percentage of migratory individuals with a high probable natal origin at a given location, as defined by the 2:1 odds ratio. The inset rose plots depict the combined probability-weighted estimates of the direction from the estimated natal origin (centre) to the capture location. Painted lady butterflies were captured during late winter/spring from **(A)** Spain (n = 13), **(B)** Morocco (n = 2), **(C)** Italy (n = 3), **(D)** Cyprus (n = 2), **(E)** Israel (n = 5), **(F)** Jordan (n = 2), **(G)** and Syria (n = 3). In the late summer/autumn, painted ladies were captured from **(H)** Senegal (n = 10), **(I)** Benin (n = 6), **(J)** Chad (n = 6), **(K)** Uganda (n = 1), and **(L)** Kenya (n = 4). Related to Figure 4.

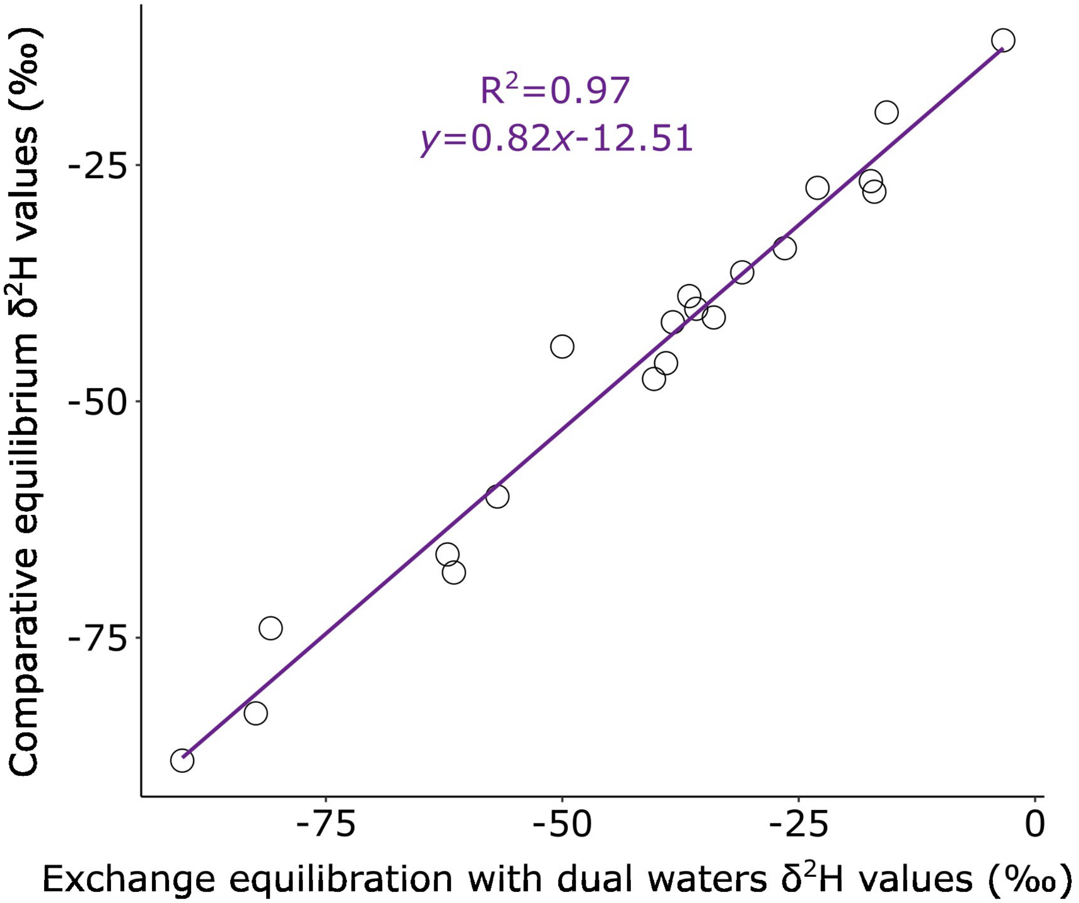

Figure S9 Scatterplot of wing samples (n = 20) from a given site used for duplicate analysis using both the exchange equilibration with dual waters (%) and comparative equilibrium protocols (%). The linear relationship was used to transform the δ^2H values of samples analysed with the exchange equilibration with dual waters protocol to align with values measured using the comparative equilibrium protocol. Related to STAR Methods.

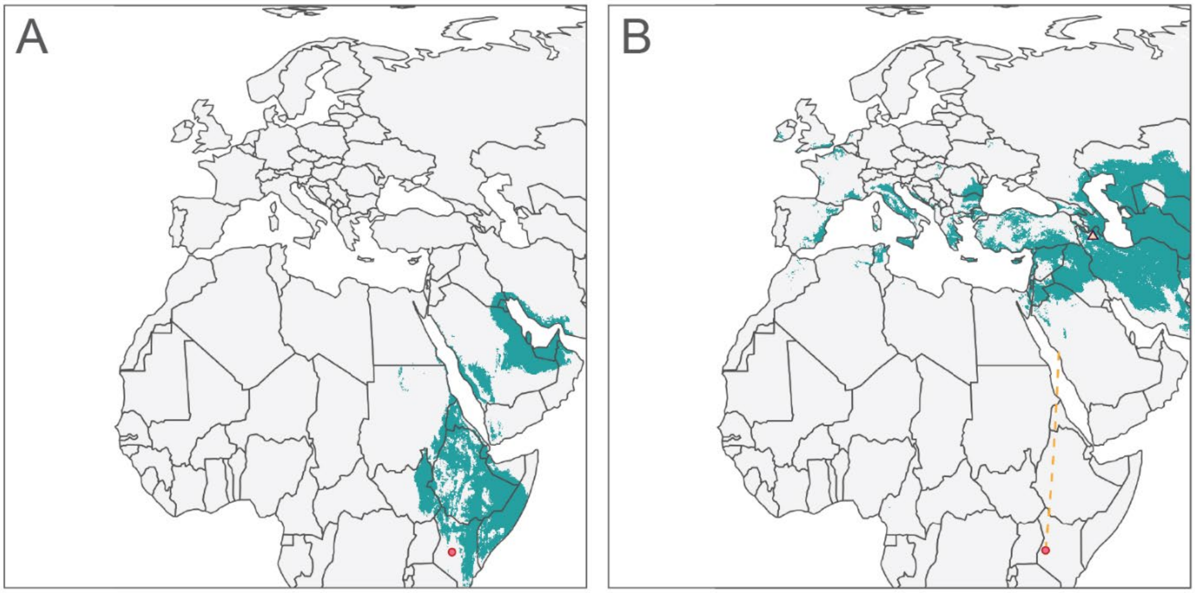

Figure S10 Examples of binary surfaces created using the 2:1 odds ratio. Teal areas indicate the highly probable area of natal origin and the red dot indicates the capture locations. **(A)** Given the proximity of the capture location to the highly probable area of natal origin (<100 km), individual GTcoll19F216 was categorized as local. **(B)** Individual RVcoll17H360 was designated as a migrant with a minimum distance (dashed yellow line) of 2,700 km. The centroid of the highly probable area of natal origin is indicated by a peach triangle. Related to STAR Methods.

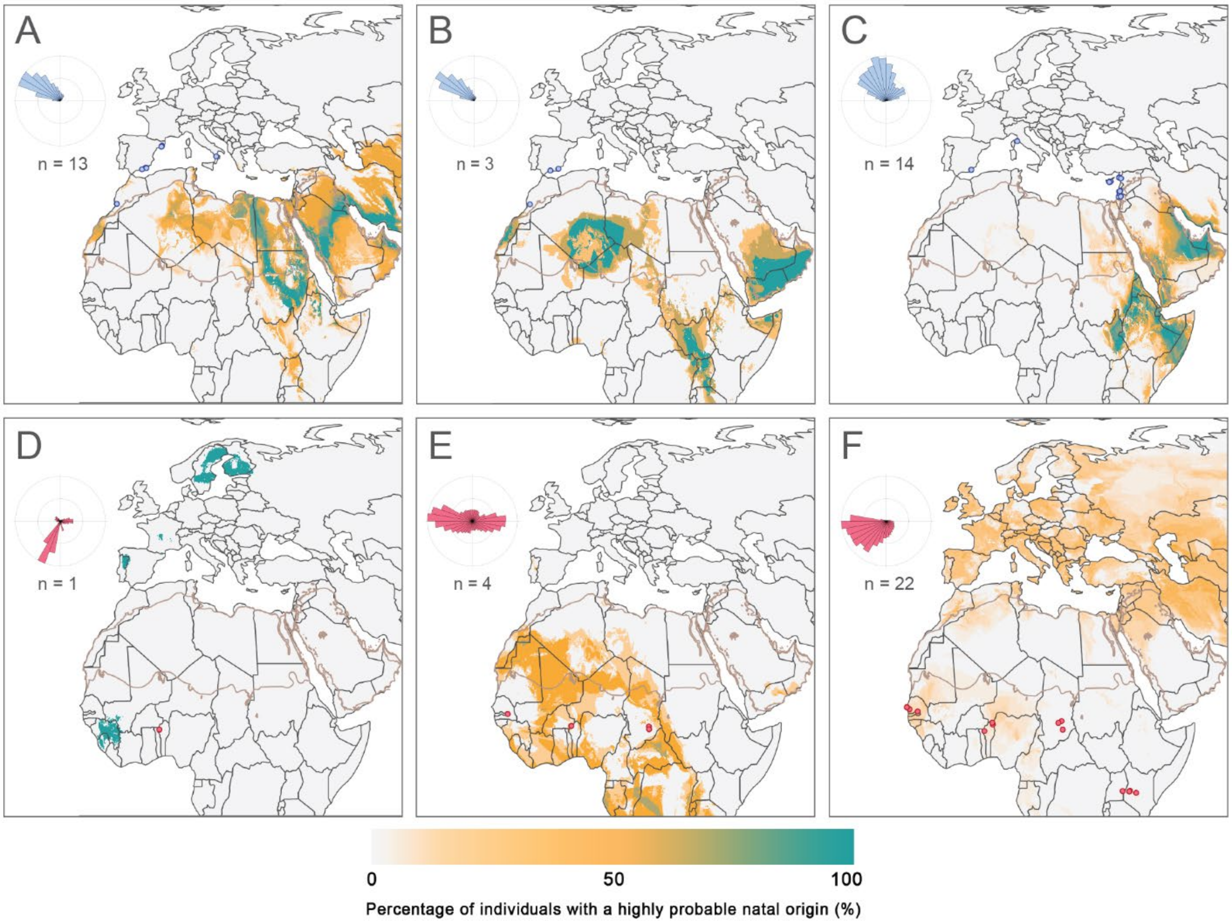

Figure S11 Stacked maps of painted ladies categorized as migratory from the δ^2H and $^{87}Sr/^{86}Sr$-based geographic assignment grouped by centroid clusters for each season, illustrating the percentage of migratory individuals with a high probable natal origin at a given location, as defined by the 2:1 odds ratio. Teal areas indicate high cohesion in the estimated area of natal origin. The inset rose plots depict the combined probability-weighted estimates of the direction from the estimated natal origin (centre) to the capture location. Captures from north of the Sahara in the late winter/spring formed three clusters made up of **(A)** 13 individuals, **(B)** three individuals, and **(C)** 14 individuals. Captures from south of the Sahara in the late summer/autumn formed three clusters made up of **(D)** one individual, **(E)** four individuals, **(F)** and 22 individuals. Related to Figure 2.

References

S1. Massicotte, P., and South, A. (2023). rnaturalearth: World Map Data from Natural Earth.

S2. Bataille, C.P., Crowley, B.E., Wooller, M.J., and Bowen, G.J. (2020). Advances in global bioavailable strontium isoscapes. Palaeogeography, Palaeoclimatology, Palaeoecology *555*, 109849. https://doi.org/10.1016/j.palaeo.2020.109849.

S3. Mooney, W.D., Laske, G., and Masters, T.G. (1998). CRUST 5.1: A global crustal model at 5° × 5°. Journal of Geophysical Research *103*, 727–747. https://doi.org/10.1029/97jb02122.

S4. Trabucco, A., and Zomer, R. (2019). Global Aridity Index and Potential Evapotranspiration (ET0) Climate Database v2. https://doi.org/10.6084/m9.figshare.7504448.v3.

S5. Hengl, T., Miller, M.A.E., Križan, J., Shepherd, K.D., Sila, A., Kilibarda, M., Antonijević, O., Glušica, L., Dobermann, A., Haefele, S.M., et al. (2021). African soil properties and nutrients mapped at 30 m spatial resolution using two-scale ensemble machine learning. Scientific Reports *11*, 1–18. https://doi.org/10.1038/s41598-021-85639-y.

S6. Chien, C., Mackey, K.R.M., Dutkiewicz, S., Mahowald, N.M., Prospero, J.M., and Paytan, A. (2016). Effects of African dust deposition on phytoplankton in the western tropical Atlantic Ocean off Barbados. Global Biogeochemical Cycles *30*, 716–734. https://doi.org/10.1002/2015GB005334.

S7. Brahney, J., Ballantyne, A.P., Kociolek, P., Leavitt, P.R., Farmer, G.L., and Neff, J.C. (2015). Ecological changes in two contrasting lakes associated with human activity and dust transport in western Wyoming: Dust-P controls on alpine lake ecology. Limnol. Oceanogr. *60*, 678–695. https://doi.org/10.1002/lno.10050.

S8. Jarvis, A., Reuter, H.I., Nelson, A., and Guevara, E. (2008). Hole-filled SRTM for the globe Version 4, available from the CGIAR-CSI SRTM 90m Database, http://srtm.csi.cgiar.org.

S9. Börker, J., Hartmann, J., Amann, T., and Romero-Mujalli, G. (2018). Terrestrial sediments of the earth: Development of a global unconsolidated sediments map database (GUM). Geochemistry, Geophysics, Geosystems *19*, 997–1024. https://doi.org/10.1002/2017GC007273.

S10. Balmino, G., Vales, N., Bonvalot, S., and Briais, A. (2012). Spherical harmonic modelling to ultra-high degree of Bouguer and isostatic anomalies. Journal of Geodesy *86*, 499–520. https://doi.org/10.1007/s00190-011-0533-4.

S11. Bataille, C.P., Brennan, S.R., Hartmann, J., Moosdorf, N., Wooller, M.J., Bowen, G.J., and Böttcher, M.E. (2014). A geostatistical framework for predicting variations in strontium concentrations and isotope ratios in Alaskan rivers. Chemical Geology *389*, 1–15. https://doi.org/10.1016/j.chemgeo.2014.08.030.

S12. Harris, I., Osborn, T.J., Jones, P., and Lister, D. (2020). Version 4 of the CRU TS monthly high-resolution gridded multivariate climate dataset. Sci Data *7*, 109. https://doi.org/10.1038/s41597-020-0453-3.

S13. Hartmann, J., and Moosdorf, N. (2012). The new global lithological map database GLiM: A representation of rock properties at the Earth surface. Geochemistry, Geophysics, Geosystems *13*. https://doi.org/10.1029/2012GC004370.

S14. Talavera, G., Bataille, C., Benyamini, D., Gascoigne-Pees, M., and Vila, R. (2018). Round-trip across the Sahara: Afrotropical painted lady butterflies recolonize the Mediterranean in early spring. Biology Letters *14*, 20180274. https://doi.org/10.1098/rsbl.2018.0274.

S15. Stefanescu, C., Soto, D.X., Talavera, G., Vila, R., and Hobson, K.A. (2016). Long-distance autumn migration across the Sahara by painted lady butterflies: exploiting resource pulses in the tropical savannah. Biology Letters *12*, 1–4. https://doi.org/10.1098/rsbl.2016.0561.

S16. Reich, M.S., Shipilina, D., Talla, V., Bahleman, F., Kébé, K., Berger, J.L., Backström, N., Talavera, G., and Bataille, C.P. (2023). Isotope geolocation and population genomics in *Vanessa cardui:* Short- and long-distance migrants are genetically undifferentiated. Preprint, https://doi.org/10.1101/2023.12.10.569105 https://doi.org/10.1101/2023.12.10.569105.
